# Does previous pregnancy experience improve folic acid supplementation uptake? A systematic review and meta-analysis

**DOI:** 10.1136/bmjopen-2025-106205

**Published:** 2026-05-04

**Authors:** Utibe Ebong, Jason J Wilson, Joanne Given, Frank Casey, Maria Loane, Helen Dolk

**Affiliations:** 1 School of Medicine, Ulster University Faculty of Life and Health Sciences, Belfast, Northern Ireland, UK; 2 School of Sport and Exercise Science, Ulster University Faculty of Life and Health Sciences, Belfast, Northern Ireland, UK; 3 Institute of Nursing and Health Research, Ulster University Faculty of Life and Health Sciences, Belfast, Northern Ireland, UK

**Keywords:** Pregnancy, Pregnant Women, PUBLIC HEALTH, Primary Health Care

## Abstract

**Abstract:**

**Objective:**

To examine whether multiparous women have more or less folic acid uptake than primiparous women.

**Outcomes:**

Preconceptional, periconceptional and postconceptional folic acid use among all pregnant women and high risk pregnant women.

**Design:**

Systematic review and meta-analysis.

**Data sources:**

MEDLINE-Ovid, CINAHL Ultimate, Scopus and ProQuest Medical.

**Eligibility criteria:**

Observational epidemiological studies comparing folic acid use between primiparous and multiparous women, published in English from January 1994 to June 2024.

**Data extraction and synthesis:**

Two reviewers screened all papers meeting the eligibility criteria. One reviewer completed data extraction and assessed study quality using an adapted version of the Newcastle-Ottawa Scale. Three other reviewers independently assessed 10% of the studies as a quality check. Random-effects (DerSimonian and Laird) meta-analyses combined results for each outcome. Heterogeneity, risk of publication bias and certainty of evidence were assessed.

**Results:**

Of the 10 982 records identified, 81 studies involving 826 855 women were included in the review. 27 studies were conducted in Europe, 22 in Asia, 11 in North America, 7 in Africa, 7 in Australia, 5 in the Middle East and 2 in South America. Multiparous women were consistently less likely to take folic acid before and during pregnancy than primiparous women. For preconceptional use, the odds were 29% lower among multiparous women (adjusted OR (aOR): 0.71; 95% CI 0.64 to 0.78; n=25 studies; I^2^=88.67%), and 58% lower in multiparous high-risk women (aOR: 0.42, 95% CI 0.27 to 0.64; n=3 studies; I^2^=27.28%). For periconceptional use, the odds were 32% lower among multiparous women (aOR: 0.68; 95% CI 0.63 to 0.74; n=28 studies; I^2^=85.46%). Postconception, the odds were 21% lower among multiparous women (aOR: 0.79; 95% CI 0.74 to 0.85; n=33 studies; I^2^=85.91%). By the second trimester, there was no significant difference between the two parity groups (aOR: 0.96; 95% CI 0.87 to 1.05; n=4 studies; I^2^=0.00%). The certainty of evidence was low for preconceptional, periconceptional and postconceptional uptake due to heterogeneity, and moderate for preconceptional uptake among high-risk women.

**Conclusions:**

Multiparous women were less likely to take folic acid preconceptional, periconceptional and postconceptionally, despite their previous pregnancy experience. Barriers to folic acid supplement uptake among multiparous women need to be identified, and strategies to address them in preconception, antenatal and interconception care should be investigated.

**PROSPERO registration number:**

CRD42024553241.

**STRENGTHS AND LIMITATIONS OF THIS STUDY**

- The review synthesised data from 826 855 women across Africa, Australia, Asia, Europe, the Middle East, North and South America, providing robust evidence.
- Only moderate and high-quality studies, assessed using the adapted Newcastle-Ottawa Scale, were included in the review.
- Significant heterogeneity was observed between studies, which downgraded the certainty of evidence for review outcomes.
- The definition of parity in the included studies may not reflect clinical definitions based on pregnancy viability.

## Introduction

 Pregnant women are advised to take folic acid supplements daily, starting before conception,^1^ to help prevent neural tube defects (NTDs).^2 3^ During fetal development, the neural tube typically closes between 21 and 28 days after conception.^2^ Sufficient folate levels are crucial for successful neural tube closure, as incomplete closure can lead to a range of NTDs, including anencephaly, encephalocele and spina bifida.^2 3^ Furthermore, there is evidence of a protective association between folic acid supplementation (FAS) and congenital heart disease,^4 5^ pre-term birth and small-for-gestational-age,^6^,^8^ autism spectrum disorder^9^,^11^ and cognitive performance.^12^,^14^ While periconceptional FAS is recommended for the prevention of NTDs and low birth weight,^6 15^ postconceptional use has been shown to improve neurodevelopment in infants.^12 13^

The WHO recommends that populations attain at least 906 nanomoles per litre of red blood cell folate to protect against NTDs.^16^ Populations with folate levels lower than this threshold are deemed to be folate insufficient. In a systematic review, Rogers *et al* found that the prevalence of folate insufficiency exceeded 40% in most countries surveyed.^17^ Despite supplementation being recommended since the 1990s,^18^,^22^ Toivonen *et al* reported variable prevalence of preconceptional folic acid supplementation across countries, with very low prevalence in African studies and higher prevalence among populations with higher educational levels.^23^ Within populations, factors such as older maternal age and pregnancy planning are known to increase women’s likelihood of taking folic acid supplements preconceptionally.^24^,^31^

To complement supplementation, many countries have introduced mandatory folate fortification for staple foods such as bread and flour.^32^ Food fortification is largely voluntary in Europe.^32^ However, the UK has mandated the fortification of bread and non-wholemeal flour from 2026.^33 34^ While food fortification has proven effective in improving folate levels and reducing the risk of NTDs,^35^,^37^ periconceptional supplementation continues to be recommended to ensure the required folate levels are attained in pregnancy.^38^

Antenatal care guidelines provide comprehensive recommendations on FAS. Women in the UK who may become pregnant, are planning to become pregnant, or are within the first 12 weeks of pregnancy are advised to take 400 mcg of folic acid each day.^15^ Women at high risk (eg, women with epilepsy, pre-gestational diabetes or a previous pregnancy with or family history of NTD) are prescribed a higher dose of folic acid (5 mg).^1 15^ Given their exposure to FAS-related information during antenatal care, women who have had previous pregnancies might be expected to have better FAS practices than first-time pregnant women. However, some studies have suggested a decline in FAS uptake as parity increased.^39^,^47^ Women at higher risk of having a child with NTD might be expected to have an even greater positive parity effect. To date, no systematic review has examined the effect of parity on FAS across populations. In this study, we systematically reviewed the evidence on the effect of parity on FAS uptake, including high-risk women. This evidence is needed to inform effective preconception, antenatal and interconception care.

## Methods

The review was reported in accordance with Preferred Reporting Items for Systematic Reviews and Meta-Analyses (PRISMA) guidelines.^48^ The protocol for this review was registered on the International Prospective Register of Systematic Review (PROSPERO), with registration number CRD42024553241.

### Review questions

1. Are multiparous women more or less likely to take folic acid in the preconceptional period (at least 1 month before conception) compared with primiparous women?
2. Are multiparous women more or less likely to take folic acid in the periconceptional period (at least 1 month before conception and continued use for at least 1 month after conception) than primiparous women?
3. Are multiparous women more or less likely to take folic acid in the postconceptional period (taking supplementation after conception) than primiparous women?
4. Are multiparous high-risk women (those with epilepsy, pre-gestational diabetes or previous infants born with NTD) more or less likely to take folic acid than primiparous high-risk pregnant women?

### Eligibility criteria

- Studies that examined FAS via tablets taken before and/or during pregnancy.
- Studies that compared FAS between primiparous and multiparous women.
- Observational (case–control, cross-sectional and cohort), hospital-based and population-based studies.
- Given the introduction of FAS guidelines in the 1990s,^18 19^ this review included published papers from 1994 to 2024.
- Articles published in English.

### Exclusion criteria

- Studies recruiting women who had never been pregnant as of the study period.
- Studies that exclusively reported serum folate levels, dietary folate intake and multivitamin intake without specifically mentioning FAS.
- Studies where only pregnant women using supplements were recruited to assess adherence, as these would not allow comparison between FAS use and non-use.
- Interventional studies, as any intervention to promote FAS uptake may introduce a deviation from pregnant women’s typical behaviour.
- Protocol papers, review papers, editorials, unpublished literature and conference abstracts.
- Studies that did not report findings by parity (the number of primiparous and multiparous women who took folic acid).
- Studies assessed to be at high risk of bias (see ‘Study quality assessment’ below).

### Information sources and search strategy

Four databases (MEDLINE-OVID, CINAHL Ultimate, Scopus and ProQuest Medical) were searched using a combination of Medical Subject Heading terms and text words (online supplemental material 1), with input from a subject librarian. The search strategy included papers published from 1994 to 2024. The initial search was performed on 24 February 2024, and an updated search was conducted on 13 June 2024. Search records were filtered for articles on human and female subjects.

### Definition of parity and gestational timing of FAS

Parity is usually defined based on clinical thresholds for pregnancy viability, where a pregnancy is considered to be viable after 20 gestational weeks.^49^ However, many of the studies reviewed did not specify their parity definition or referred to previous pregnancies rather than parity. This review used parity as defined in the included studies.

Studies were grouped into three categories based on the timing of supplementation, with some studies appearing in more than one outcome category:

- Preconceptional FAS: Referring to studies that examined preconceptional intake. These studies did not specify whether FAS continued postconception.
- Periconceptional FAS: Referring to studies where intake started preconception and continued postconception (see table 1).
- Postconceptional FAS: Referring to studies that investigated postconceptional intake (regardless of when intake was initiated), which were further divided into the first and later trimesters.

**Table 1:** Definitions of preconception and periconception adopted by individual studies

Timing of folic acid intake	Number of studies
Definitions of preconception	**25**
1 month before conception	2
3 months before conception	5
3 or 1 month/s before conception	1
Before conception	17
Definitions of periconception	**29**
1 month preconception and continued use 1 month postconception	1
1 month preconception and continued use 2 months postconception	3
1 month preconception and continued use 3 months postconception	8
1 month preconception and continued use 4 months postconception	1
1 month preconception and continued use postconception	1
2 months preconception and continued use 1 month postconception	1
2 months preconception and continued use 3 months postconception	2
3 months preconception and continued use 1 month postconception	1
3 months preconception and continued use 3 months postconception	3
3 months preconception and continued use postconception	1
3 months preconception or first trimester	1
3 months preconception or within 1 month after conception	1
Before conception and continued use 2 months postconception	1
Before conception and continued use postconception	2
Before conception and continued use 3 months postconception	2

### Selection process

UE executed the predefined search strategy in each of the specified sources. UE and JW independently completed the title and abstract screening of the returned records as well as the full-text screening of eligible records. Inconsistencies between these independent screenings were discussed and resolved jointly. When conflicts in screening could not be resolved, HD, ML and JG reviewed and resolved them. The web-based systematic review management programme, Covidence, was used to manage the search results, including removing duplicate records and managing independent record screening.

### Data extraction

UE extracted data using the following headings: country, study period, study setting, study design, data collection method, sample size and FAS outcomes (online supplemental material 2).

### Study quality assessment

UE assessed the risk of bias in the eligible studies. HD, ML and JG independently assessed a random subset of the studies (10%) to ensure reliability and accuracy—two to four studies each. UE adapted the Newcastle-Ottawa Scale (NOS) to determine the risk of bias in the studies included in this review (online supplemental material 3). The NOS tool scores studies based on three primary domains: the selection of study group(s), the comparability of the study group(s) and the ascertainment of the outcome, with a maximum score of 9 points.^50^ This review used a benchmark of four points for moderate quality and seven points for high quality, in line with recent practice.^51^,^54^ The summary of the risk of bias assessment is presented in online supplemental material 4.

### Statistical analysis

Several included studies reported ORs for multiple multiparous subgroups (eg, second birth, third or more births) rather than a single multiparous category. To make these results comparable to studies with a single multiparous category, we combined the subgroup-specific ORs within each study using an inverse-variance weighting method on the log scale.^55^,^57^

Meta-analysis was performed using the random-effects (DerSimonian and Laird) method to allow for heterogeneity.^58^ The meta-analysis included adjusted and unadjusted ORs, where adjusted ORs (aORs) were not reported. Where included studies reported both unadjusted ORs and aORs, aORs were preferred for meta-analysis in accordance with guidelines.^59^ Further, a sensitivity analysis was conducted using only studies with aORs.

Heterogeneity was assessed using Cochrane’s Q test (with p<0.05 indicating statistically significant heterogeneity) and quantified using the I² statistic, representing the proportion of variability attributable to between-study differences rather than chance. Where substantial heterogeneity was observed, subgroup analyses were conducted to explore potential sources of heterogeneity based on study-level characteristics (eg, study design, geographical region, study population). Differences in pooled estimates between subgroups were also reported to assess any changes in the magnitude or direction of the observed effect due to study-level characteristics.

### Publication bias

Publication bias was assessed using the funnel plot in Stata V.18.^60^ In addition, Egger’s tests were used to check publication bias. A p value<0.05 inferred possible publication bias or bias due to missing data.

### Certainty of evidence

The Grading of Recommendations, Assessment, Development and Evaluation (GRADE) guideline development tool (GRADEpro GDT) was used to assess the quality of evidence on the effect of parity on the FAS outcomes. The GRADE approach categorises the quality of evidence into high, moderate, low and very low quality.^61^

## Results

### Study selection

**Figure 1:**
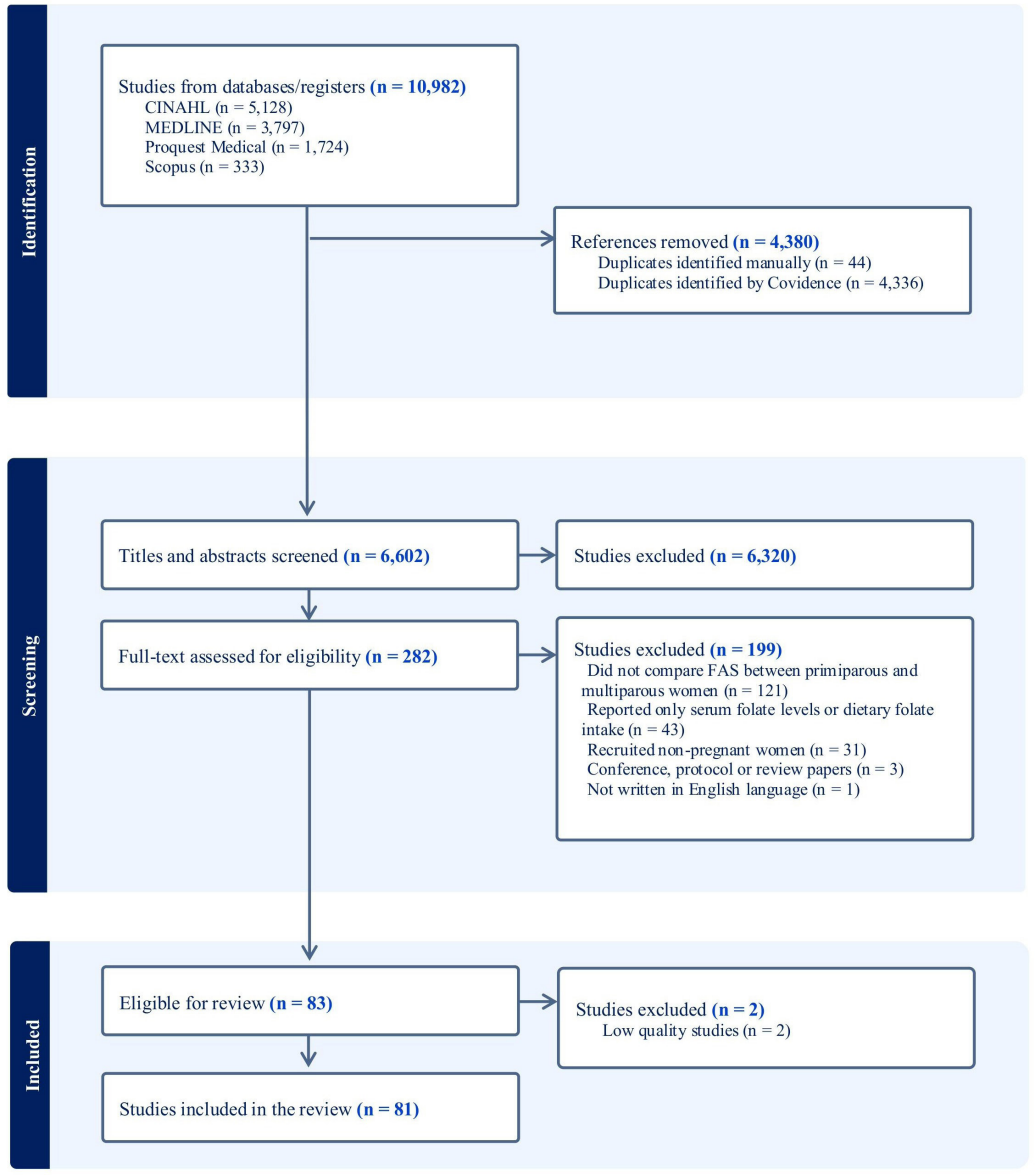
PRISMA flow chart illustrating the inclusion process of studies for the review. FAS, folic acid supplementation; PRISMA, Preferred Reporting Items for Systematic Reviews and Meta-Analyses.

The search strategy returned 10 982 records from all four sources, of which 83 studies were eligible for review. 2 studies were further excluded due to low quality,^62 63^ giving a final total of 81 studies included in this review (figure 1).

### Study characteristics

**Figure 2:**
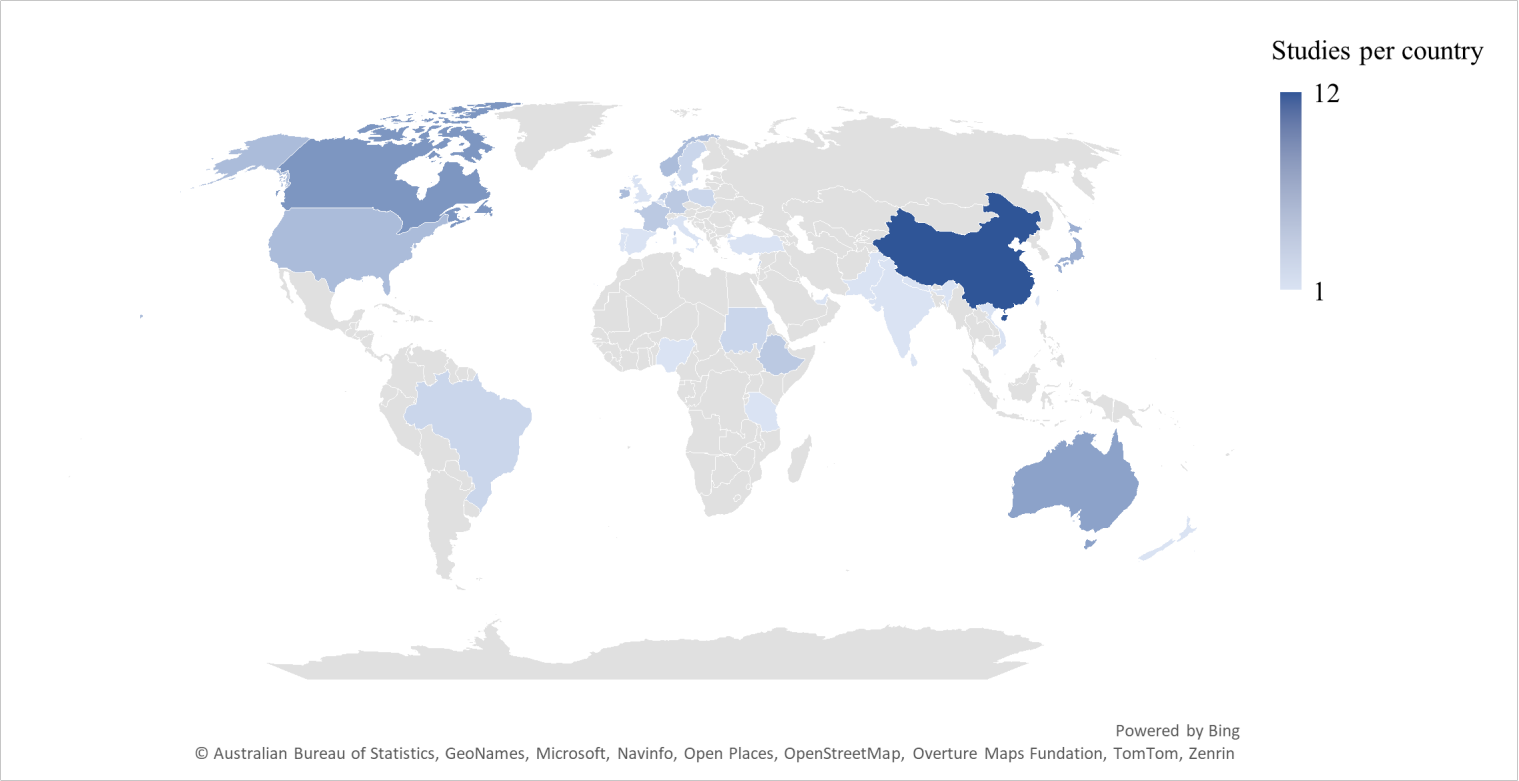
Map showing the location of studies included in this systematic review.

The study period ranged from 2002 to 2024. 27 (33%) of the studies were conducted in Europe, 22 (27%) in Asia, 11 (14%) in North America, 7 (9%) in Africa, 7 (9%) in Australia, 5 (6%) in the Middle East and 2 (2%) in South America (figure 2 and online supplemental material 2).

Sample sizes ranged from 140 to 197 346, involving a total of 826 855 women across 33 countries. Regarding study settings, 33 (41%) of these studies were hospital-based and 48 (59%) were population-based where participants were recruited from communities or national registers/databases. 53 of the 81 studies recruited pregnant women, whereas 31% recruited postpartum women who were retrospectively interviewed about their FAS practices. A small number of studies (4%) recruited both pregnant and postpartum women.

Three studies sampled women with a high risk of having children with congenital anomalies. Two of the three studies recruited pregnant women with epilepsy.^64 65^ The other study analysed records of pregnant women with conditions likely to increase the risk of congenital anomalies.^66^

Of the 81 studies, 59 (73%) were cross-sectional, 17 (21%) were cohort and 5 (6%) were case–control studies. The cohort studies included in this review were designed to investigate associations between environmental/personal characteristics (including FAS) and health outcomes, including gestational diabetes mellitus,^26 67^ pre-eclampsia,^68^ fetal alcohol spectrum disorders,^45^ chronic diseases (cardiovascular diseases, cancers, diabetes, hypertension, etc)^69^ and child health and development.^2546 70^,^75^ Three of the five case–control studies reported data from the control group.^76^,^78^ Two case–control studies (cases were mothers of infants with birth defects in one study, and in the other, cases were mothers of infants born preterm) reported the combined data from cases and controls.^27 79^ Parity was examined as one of the many socio-demographic determinants of folic acid uptake in some of the included studies. In others, it was merely reported as one of the analysis outputs, but its effect was not discussed. Further, eight of the included studies reported more than one FAS outcome (online supplemental material 2).

Individual studies adopted different definitions for preconception and periconception, reflecting the disparity in FAS guidelines and recommendations across countries. Regarding preconceptional intake, 68% of preconceptional studies did not stipulate a time frame and simply defined it as ‘use before conception’ (table 1). For periconception, the most common definition adopted was 1 month use preconception and continued use 3 months postconception (table 1):

### Risk of bias in studies

Of the 81 studies, 30 studies were assessed as having moderate quality and 51 as having high quality (online supplemental material 4).

### Results of syntheses

#### Overall intake

**Figure 3:**
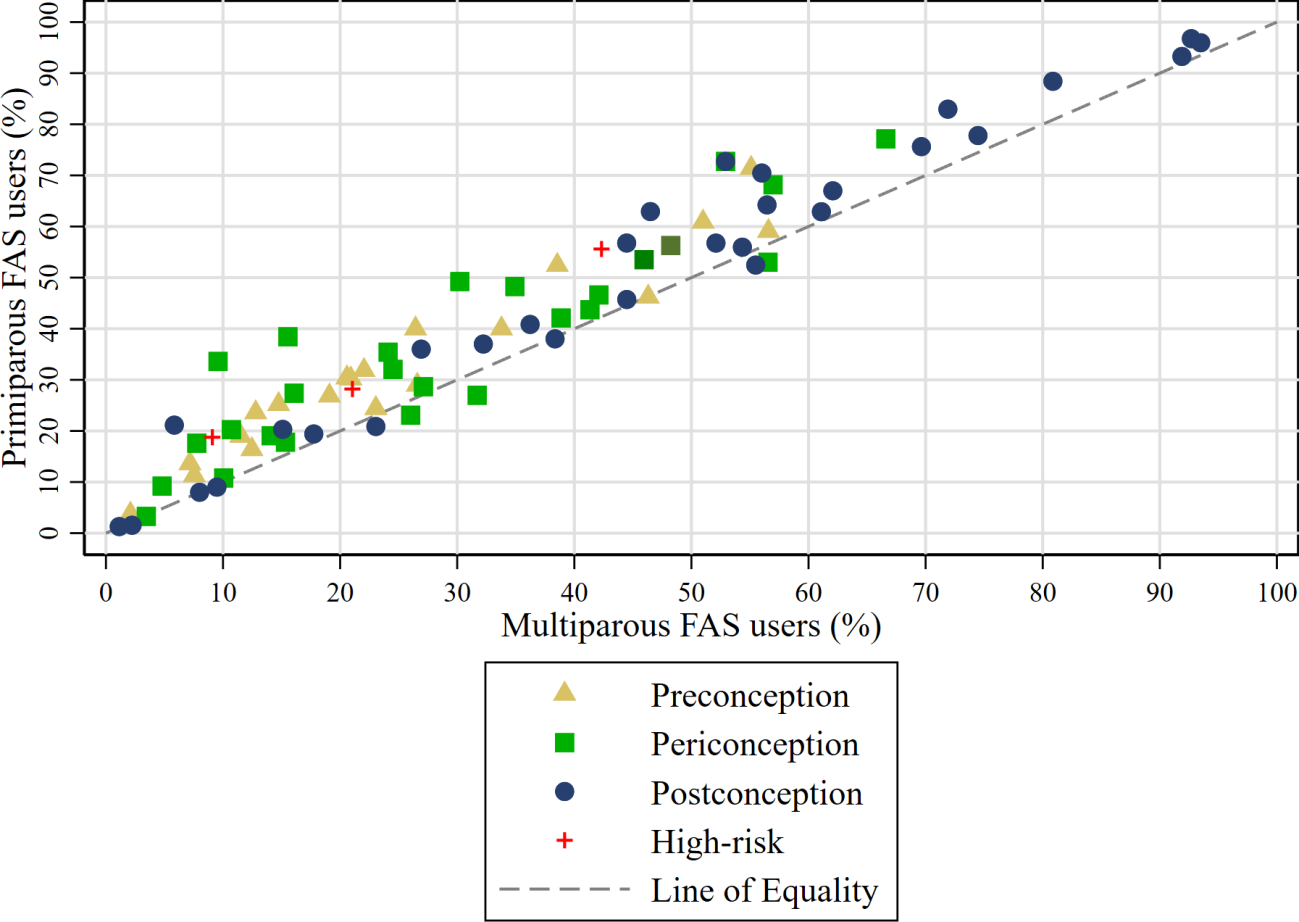
FAS intake in primiparous and multiparous women by study, and by timing of intake. FAS, folic acid supplementation.

The reported prevalence of folic acid intake varied across studies, ranging from 1.2% in Sweden^80^ to 92.1% in Sudan.^40^ A visual inspection of figure 3 shows most study markers were above the line of equality, indicating that FAS was more common among primiparous than multiparous women, regardless of whether it occurred preconception, periconception or postconception and regardless of whether overall FAS intake was high or low.

#### Preconceptional intake

**Figure 4:**
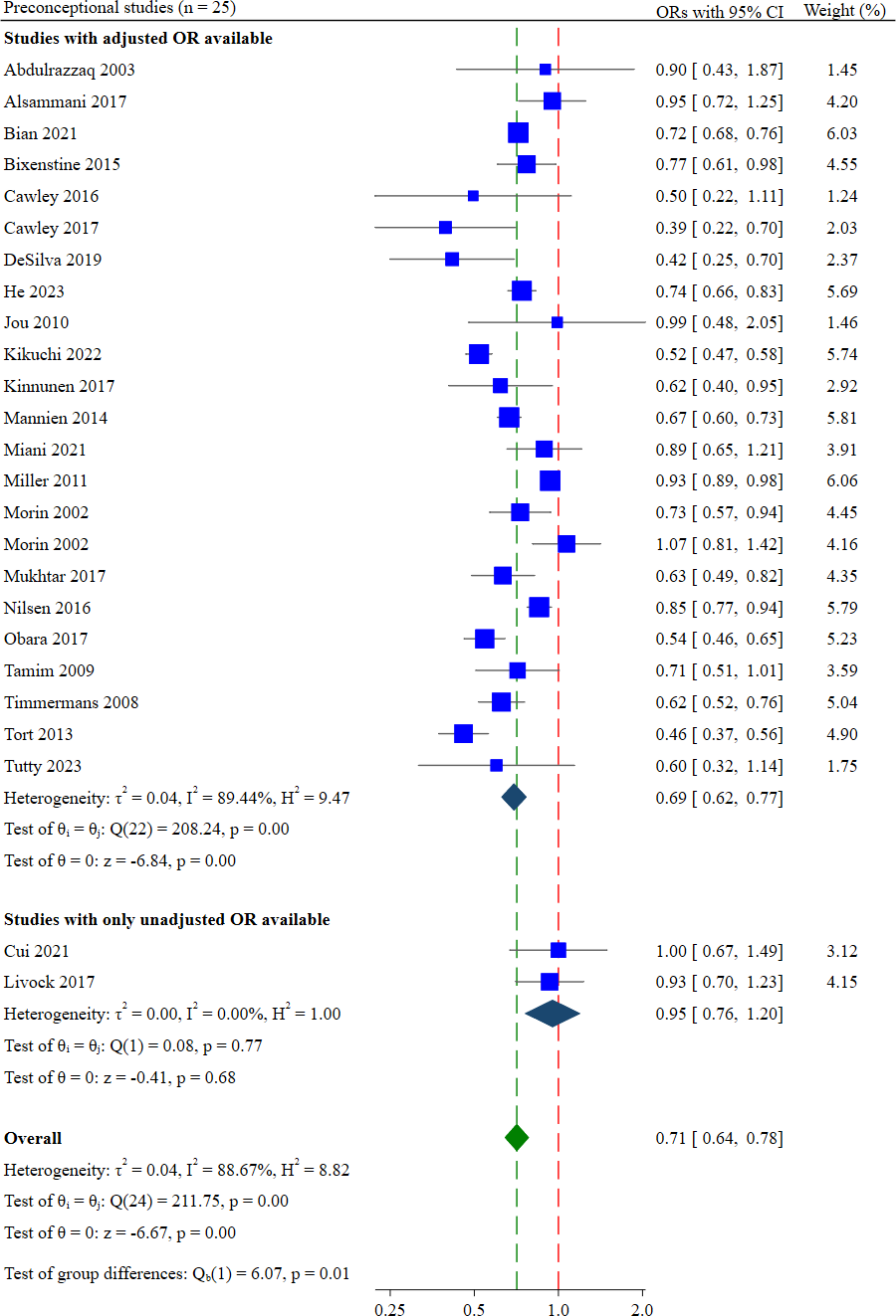
Forest plot showing the effect estimates for preconceptional supplementation.

25 studies examined preconceptional FAS.^2529 42 44^,^46 67 81^ Pooling these preconceptional studies, the odds of supplementation were 29% lower among multiparous women (aOR: 0.71; 95% CI 0.64 to 0.79; low certainty of evidence; figure 4) compared with primiparous women. The absolute effect of multiparity on preconceptional intake was a difference of 8 per 100 women compared with primiparous women (table 2).

**Table 2:** GRADE summary of findings^177^

Outcomes	Patients (studies), N	Relative effect (95% CI)	Absolute effects (95% CI)			Certainty
			Primiparous women	Multiparous women	Difference	
Preconception	266 423 (25 non-randomised studies)	OR=0.71 (0.64 to 0.78)	38 per 100	31 per 100 (33 to 28)	8 fewer per 100 (from 10 fewer to 6 fewer)	⨁⨁◯◯ Low*
Periconception	162 759 (29 non-randomised studies)	OR=0.69 (0.64 to 0.76)	31 per 100	24 per 100 (25 to 22)	7 fewer per 100 (from 9 fewer to 6 fewer)	⨁⨁◯◯ Low*
Postconception	840 979 (34 non-randomised studies)	OR=0.80 (0.74 to 0.85)	11 per 100	9 per 100 (9 to 8)	2 fewer per 100 (from 3 fewer to 1 fewer)	⨁⨁◯◯ Low*
Preconception among high-risk women	2047 (3 non-randomised studies)	OR=0.42 (0.27 to 0.64)	39 per 100	21 per 100 (29 to 15)	18 fewer per 100 (from 24 fewer to 10 fewer)	⨁⨁⨁◯ Moderate

* Some unexplained heterogeneity was observed between included studies.

GRADE, Grading of Recommendations Assessment, Development and Evaluation.

Significant heterogeneity was observed across these 25 studies (Cochrane’s Q test=211.75, p<0.001, I^2^=88.67%). Heterogeneity was lower when studies were grouped by study locations (studies conducted in Australia: aOR: 0.83; 95% CI 0.56 to 1.21, Cochrane’s Q test=1.50, p=0.22, I^2^=33.24%, n=2 and studies conducted in the Middle East: aOR: 0.74; 95% CI 0.55 to 1.02, Cochrane’s Q test=0.31, p=0.58, I^2^=0.00%, n=2). The difference in pooled ORs between these study location subgroups was not significant (test of group difference=9.80, p=0.08; online supplemental material 5).

23 of the 25 studies adjusted for confounders, most frequently maternal age, education, socioeconomic status, smoking, relationship status and pregnancy planning. A sensitivity analysis restricted to these 23 studies found that the odds were 31% lower among multiparous than primiparous women (aOR: 0.69; 95% CI 0.62 to 0.77, n=23 studies, I^2^=89.44%).

#### Periconceptional intake

**Figure 5:**
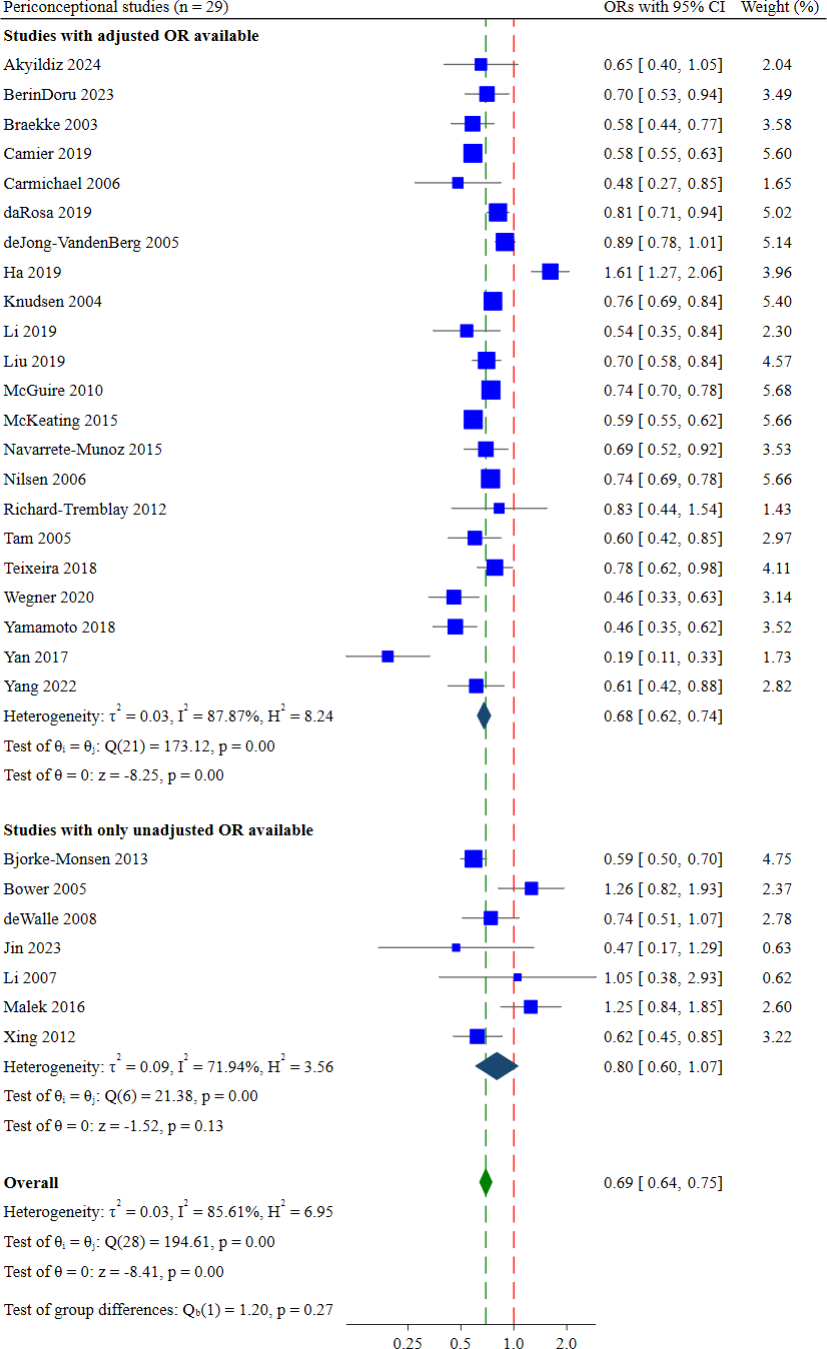
Forest plot showing the effect estimates for periconceptional supplementation.

29 studies examined periconceptional FAS.^2426^,^28 30 31 39 41 70 71 73^ Pooling these studies, the odds of supplementation were 21% lower among multiparous women (aOR: 0.69; 95% CI 0.64 to 0.76, low certainty of evidence; figure 5) than primiparous women. The absolute effect of multiparity on periconceptional intake was a difference of 7 per 100 women compared with primiparous women (table 2).

Heterogeneity was high among these 29 studies (Cochrane’s Q test=194.61, p<0.001, I^2^=85.61%). Heterogeneity was reduced when studies were grouped by food fortification policies (mandatory fortification: aOR: 0.82; 95% CI 0.72 to 0.93, Cochrane’s Q test=4.57, p=0.21, I^2^=34.28%, n=4 and fortification policy not reported: aOR: 0.69; 95% CI 0.62 to 0.77, Cochrane’s Q test=21.10, p=0.03, I^2^=47.87%, n=12). The test for differences between these fortification subgroups indicated borderline differences in the pooled ORs (test of group difference=7.62, p=0.05; online supplemental material 6).

22 of the 29 studies adjusted for confounders, most frequently maternal age, education, socioeconomic status, smoking, relationship status and pregnancy planning. A sensitivity analysis restricted to these 22 studies found similar odds among multiparous women (aOR: 0.68; 95% CI 0.62 to 0.74, n=22 studies, I^2^=87.87%).

#### Postconceptional intake

**Figure 6:**
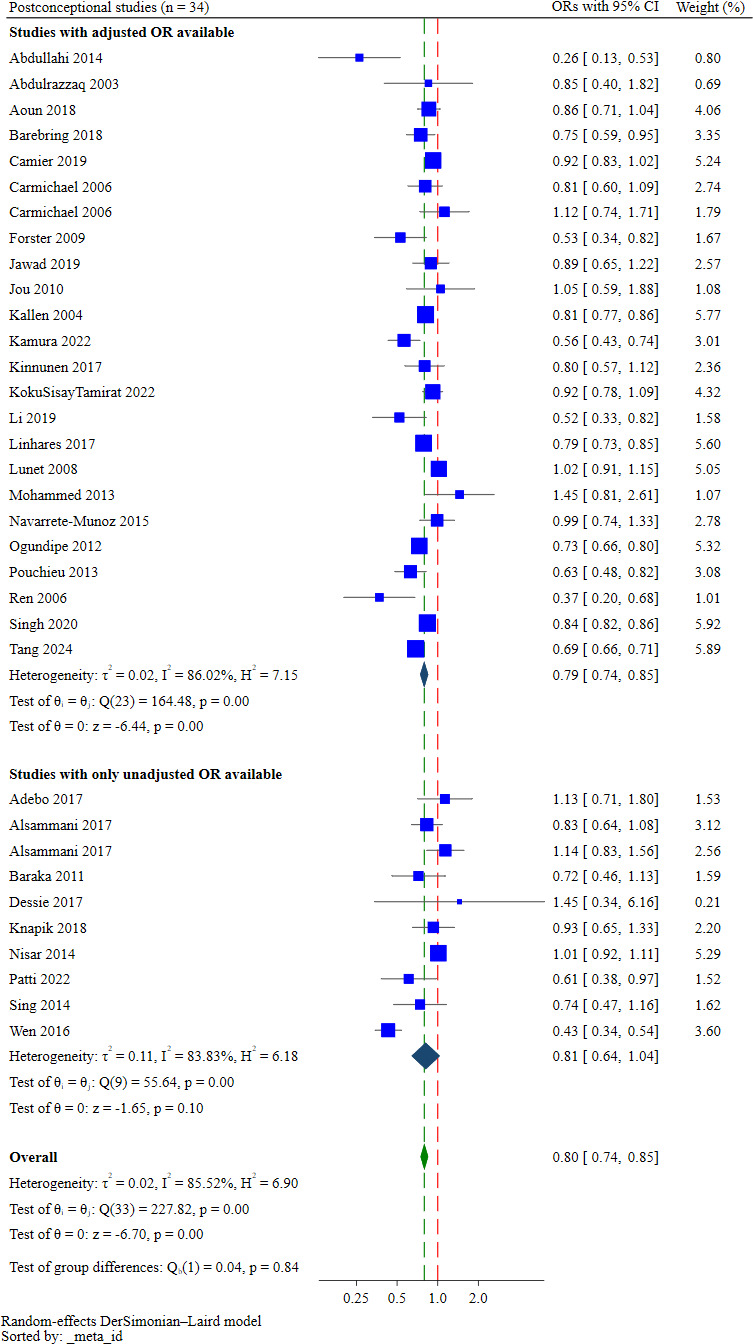
Forest plot showing the pooled effect estimates for postconceptional supplementation.

34 studies examined postconceptional FAS.^4043 47 62 67^,^69 71 72 77 79^ Pooling these studies, the odds of supplementation were 20% lower among multiparous women (aOR: 0.80; 95% CI 0.74 to 0.85, low certainty of evidence; figure 6) than primiparous women. Grouping the studies by trimester, multiparous women were less likely than primiparous women to take folic acid in the first trimester (aOR: 0.73; 95% CI 0.65 to 0.82, n=10 studies, I^2^=78.86%). By the second and third trimesters, the difference in intake between the parity groups was not statistically significant (aOR: 0.96; 95% CI 0.87 to 1.05, n=4 studies, I^2^=0.00%).

The absolute effect of multiparity on postconceptional intake was a difference of 2 per 100 women compared with primiparous women (table 2). The low absolute effects on postconceptional intake were skewed by one study in Sweden,^80^ which contributed 69% of the sample for postconceptional intake and reported exceptionally low FAS uptake (1.2% of the sample). This, however, does not impact the pooled OR as study weights in the meta-analysis are determined by the precision of the effect size. Consequently, the absolute effects for postconceptional intake should be interpreted with caution.

Heterogeneity was high among these 34 studies (Cochrane’s Q test=227.82, p<0.001, I^2^=85.52%). Heterogeneity was reduced when studies were grouped by food fortification policies (no fortification: aOR: 0.91; 95% CI 0.77 to 1.07, Cochrane’s Q test=1.40, p=0.49, I^2^=0.00%, n=3 and mandatory fortification: aOR: 0.69; 95% CI 0.66 to 0.71, Cochrane’s Q test=0.24, p=0.62, I^2^=0.00%, n=2). The pooled ORs differed significantly between these fortification subgroups (test of group difference=25.54, p<0.001; online supplemental material 7).

24 of the 34 studies adjusted for confounders, most frequently maternal age, education, socioeconomic status, smoking, relationship status and place of residence (urban vs rural). A sensitivity analysis restricted to these 24 studies found similar odds among multiparous women (aOR: 0.79; 95% CI 0.74 to 0.85, n=24 studies, I^2^=86.02%).

#### Folic acid (preconceptional) uptake among high-risk women

**Figure 7:**
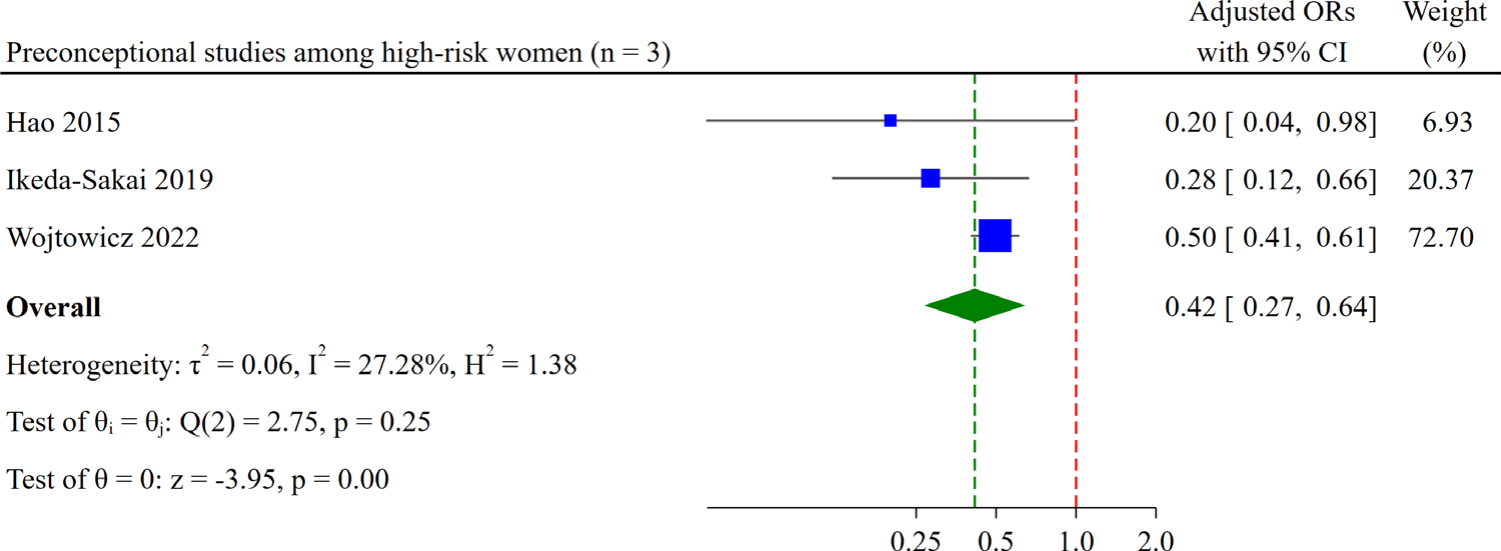
Forest plot showing pooled effect estimates for preconceptional folic acid uptake among high-risk women.

Three studies examined folic acid intake among women with epilepsy and those at risk of having children with congenital anomalies.^64^,^66^ The odds of supplementation were 58% lower among multiparous women (aOR: 0.42, 95% CI 0.27 to 0.64, n=3 studies, moderate certainty of evidence; figure 7) than primiparous women. In absolute terms, multiparity accounted for 18 fewer high-risk women per 100, taking folic acid preconceptionally. Maternal age, smoking and pregnancy planning were the most frequently adjusted confounders among these three studies. Heterogeneity was negligible among these studies (Cochrane’s Q test=2.75, p=0.25, I^2^=27.28%).

### Publication bias

A visual inspection of the funnel plots showed clustered study markers at the top of the funnels, suggesting high precision in the effect estimates. The distribution of study markers for preconception and periconception FAS showed visual asymmetry, suggesting potential bias (online supplemental material 8). The Egger’s regression test quantitatively confirmed that publication bias was not detected (p>0.05) across studies (table 3).

**Table 3:** Egger’s regression test outputs

FAS outcomes	Number of studies	Bias coefficient	t value	P value
Preconception	25	−0.28	−0.48	0.63
Periconception	29	−0.51	−1.00	0.33
Postconception	34	−0.38	−0.95	0.35
High-risk women	3	−1.46	−1.64	0.35

FAS, folic acid supplementation.

### Certainty of evidence

The certainty of evidence was low for preconceptional, periconceptional and postconceptional FAS, due to the heterogeneity between studies. The certainty of evidence was moderate for preconceptional intake among high-risk pregnant women.

## Discussion

### Principal findings

This is the only systematic review that has focused on parity as a determinant of FAS pre, peri and postconceptionally. While many individual studies reported a parity effect, its implications were rarely commented on.^7577 78 82^,^85 99^ We found that multiparous women were consistently less likely than primiparous women to take folic acid from preconception to postconception, despite exposure to previous antenatal care. This suggests that previous pregnancy experience has not improved FAS practices among multiparous women.

Our findings also highlight that even for women at high risk of NTDs, for whom higher doses of FAS are recommended, multiparous women are less likely than primiparous women to use FAS, which is clinically relevant given the overall low uptake of FAS among this high-risk group.^64 65^

In this review, publication bias was not detected across the assessed outcomes. However, the certainty of evidence was limited by substantial heterogeneity for three of the four outcomes. Only one outcome (preconceptional uptake among high risk women) achieved a moderate level of certainty. While these findings indicate a clear effect of multiparity on folic acid uptake, this should be interpreted with caution due to methodological and clinical differences between the included studies.

### Preconceptional and periconceptional supplementation

Studies of the barriers to uptake of FAS among pregnant women have cited a lack of knowledge/awareness,^31 127^ unplanned pregnancies,^24 31 128^ low socioeconomic status^24 129 130^ and low educational attainment.^31 105 128 130^ Multiparous women have cited childcare responsibilities as a reason for suboptimal FAS,^45 131^ and many studies have shown that multiparous women have higher rates of unplanned pregnancy.^132^,^134^ In addition, a healthy outcome from the previous pregnancy has been suggested to lower motivation or belief that folic acid is necessary.^31 39 41 44 67^ Multiparous women have also reported the belief that folic acid caused nausea during a previous pregnancy.^131 135^ This perception may lead to their discontinuation of FAS in the subsequent pregnancy.

Behaviour change theories, such as the Capability-Opportunity-Motivation Behaviour (COM-B) framework, emphasise the importance of capability (physical and psychological), opportunity (physical and social) and motivation (automatic and reflective) in changing or maintaining behaviour.^136 137^ Research has largely focused on knowledge and awareness of folic acid,^138^ which can increase capability and motivation, but is not in itself enough to change behaviour. All three components—capability, opportunity and motivation—must be present for behaviour to change.^139 140^

### Postconceptional supplementation

Evidence from this review shows that multiparous women, overall, had significantly lower odds of taking folic acid postconceptionally, compared with primiparous women. However, by the second and third trimesters, there was no significant difference in uptake between primiparous and multiparous women. By the second trimester, it is expected that almost all pregnant women will have begun antenatal appointments, during which the need for supplementation would be emphasised. This may explain why the supplementation rates between primiparous and multiparous women were similar at this stage of the pregnancy. However, these women would have missed the critical window for supplementation needed to protect against NTDs.

### Folic acid use among high-risk pregnant women

Our findings highlight that among women at higher risk of having a baby with NTDs, multiparous women are less likely than primiparous women to use FAS, at any dose, preconceptionally. While high-risk women are advised to take a high dose of folic acid (5 mg), only one of the three studies specified the FAS dosage.^64^ Indeed, some studies have questioned the benefits of high dose folic acid for high-risk pregnant women compared with the normal (400 mcg) dose.^141 142^ The safety of high dose folic acid has also been investigated.^143^,^145^ Nonetheless, there is clear evidence of the importance of early initiation and uptake of folic acid supplements in preventing NTDs, particularly among this high-risk group of women.

Other studies have reported low FAS prevalence among high-risk women but have not investigated the role of parity.^146^,^151^ The low prevalence in this high-risk group is surprising, as these women would be expected to have more exposure to specialised care as per WHO guidelines.^152^ Studies have highlighted gaps in the recommended specialised care for high-risk pregnancies.^153 154^ Pregnancy planning and the type of care received by these high-risk women could determine whether or not folic acid is initiated preconceptionally. Studies have shown that women at high risk who planned their pregnancies were more likely to take folic acid compared with those who did not.^147 155^ Among those who were sexually active but not planning to get pregnant, visits to specialist gynaecology care were a key enabler for FAS.^64 147^ On the other hand, visits to their neurologist and primary care provider did not impact FAS practices.^64 147^ This suggests that some specialists may be promoting periconceptional supplementation more effectively than others. It is possible that a lack of coordination between specialists can lead to the omission of key messages about folic acid.^156^

### Implications for clinicians and policymakers

Multiparous women almost universally have experience of antenatal care from their previous pregnancy. In some countries, interconception care is available to women during the interpregnancy interval. The results of our review suggest that these pregnancy-related services are failing to improve, or even maintain, levels of pre and periconceptional FAS in future pregnancies.

While the COM-B framework has been used to guide behaviour change interventions for pregnant women in other areas, such as smoking,^157^ physical activity^137 158^ and diet,^136 159^ only one study has applied it to folic acid.^138^ Evidence from our review suggests that knowledge and awareness of FAS needed to be improved, but other aspects, such as motivation and opportunity, also needed improvement. Meta-analyses show low utilisation of preconception^160 161^ and interconception care,^162^ thereby creating the first barrier to improvements in these COM-B components. Preconception care before the first pregnancy, antenatal care during the first pregnancy and interconception care after the first pregnancy could look ahead to subsequent pregnancies, emphasising the importance of initiating FAS preconceptionally.^163^,^165^ This could strengthen women’s capability to supplement periconceptionally. Consideration could be given to the role of the partner and other social support,^157 159^ including community-based support groups, to enable the required social opportunity for periconceptional supplementation. In terms of physical opportunity, policy changes such as provision of free folic acid supplements and using a more diverse distribution network,^138 166 167^ may improve FAS uptake. Negative beliefs and perceptions can limit women’s motivation to change behaviour,^137 138^ hence it is important that health information provided by clinicians and others address known misconceptions about FAS to improve women’s capability and motivation. The implications for workforce training and the need to include time for folic acid-related interventions in preconception, antenatal and interconception care need to be considered.

For high-risk women, there is a particular need for coordination among services such as primary care, obstetrics and other specialist services (eg, for diabetes and epilepsy) to emphasise the need to take folic acid supplements before each pregnancy, thereby improving women’s capability, motivation and opportunity. For women with conditions such as diabetes and epilepsy, preconception care is recommended to ensure that the condition and treatment are regulated before pregnancy,^168^,^171^ FAS could be improved among this high-risk group if preconception care is optimally designed, available and used.^160^

Our findings reinforce the importance of mandatory folic acid fortification as implemented in many countries,^32 172 173^ and soon to be introduced in the UK.^33 34^ This will reach primiparous and multiparous women alike, but efforts will still be needed to improve supplementation uptake, especially among women who do not routinely consume the fortified food types.^174^ Recently, in the USA, California state laws have extended fortification to culturally-specific staples such as tacos and tortillas, to protect Latino communities from the risk of NTDs.^175^

### Strengths and limitations of the systematic review

This review used a robust systematic review methodology, synthesising data from 81 studies covering 826 855 women in 33 countries and 7 world regions. Other strengths include adapting the NOS tool to assess the risk of bias, including how folic acid use was ascertained and excluding low-quality studies from data synthesis to strengthen the quality of evidence. Only moderate and high-quality studies from the risk of bias assessment were included in the review. While risk of bias assessment was conducted by one reviewer, a random subset (10%) of these studies were independently assessed by three other reviewers to ensure reliability and accuracy.

We used adjusted effect estimates where available. While studies varied in the covariates included in the adjusted analysis, maternal age and pregnancy planning were adjusted for in most studies. We can be confident that the multiparity effect is independent of the effect of maternal age and largely independent of the effect of pregnancy planning.

The ascertainment of FAS uptake may depend on the sources of data used. However, FAS uptake was ascertained in the same way for primiparous and multiparous women, therefore ascertainment bias on the effect estimate is expected to be minimal. Differences in FAS ascertainment can affect the FAS prevalence estimates for different populations, and therefore caution is needed in interpreting the absolute effect by parity.

Heterogeneity downgraded the certainty of evidence for three of the FAS outcomes but this can be expected in observational studies of behavioural factors. The populations studied differed socio-demographically, in their healthcare systems, methods of ascertaining FAS and country-specific recommendations regarding FAS. The observed heterogeneity in the syntheses was, therefore, to be expected. This also means that while we give summary estimates of FAS uptake for all studies pooled to show the extent of the parity effect, these estimates may not apply to all populations.

The assessment of high-risk women was limited by the small number of studies assessing FAS by parity among high-risk women (n=3 studies). Another limitation of this review was the restriction of the search strategy to publications in English. Further, this review used the parity definition in the included studies; however, it is possible that study definitions do not align with existing clinical definitions of parity.^49 176^ This meant that in some studies, multiparous women may have included women whose previous pregnancy(ies) did not attain viability. This is important in the context of experiencing full antenatal care and folic acid knowledge/use in their previous pregnancy(ies).

## Conclusion

This review highlights that multiparous women are consistently less likely to use folic acid before or during the first trimester of pregnancy. High-risk multiparous women were also found to have lower adherence to FAS despite their increased risk of NTDs. Barriers to adequate supplementation among multiparous women need to be further investigated. Preconception, antenatal and interconception care are crucial to promoting adequate supplementation and reducing the burden of NTDs.

Future research should explore interventions to promote preconceptional supplementation using the COM-B model of behaviour change, which recognises capability, motivation and opportunity as essential interacting components.

## Other information

### Registration and protocol

Results are reported following the PRISMA 2020 guideline.^48^

Some amendments were made to the review compared with the protocol at the point of registration. First, folic acid use among high-risk women was not specified as an outcome at the point of registering the protocol. The decision to include this outcome was made at the point of data extraction, when studies among high-risk women were identified. Being a particular subgroup of pregnant women, this outcome was then introduced into the review to allow a separate discussion of findings.

Lastly, the registered protocol specified a systematic review without meta-analysis, considering the variations in study populations, methods of assessing folic acid intake and definitions for preconception and periconception. However, the analysis of studies by subgroups (pre, peri and postconception) allowed for pooled synthesis while acknowledging the heterogeneity between studies as one of the review’s limitations.

## Supplementary material

10.1136/bmjopen-2025-106205 online supplemental file 1

## Data Availability

All data relevant to the study are included in the article or uploaded as supplementary information.
