# Supplementary material for: Does previous pregnancy experience improve folic acid supplementation uptake? A systematic review and meta-analysis
Source: BMJ Open. 2026 May 4;16(5):e106205. doi: 10.1136/bmjopen-2025-106205 (PMC13141133; doi:10.1136/bmjopen-2025-106205)

**SUPPLEMENTARY MATERIAL 1: SEARCH STRATEGY**

Databases searched were: Medline-OVID, CINAHL Ultimate, SCOPUS and Proquest Medical.

The following search strategies were used:

1. **Medline-OVID:**
2. pregnan*.mp. [mp=title, book title, abstract, original title, name of substance word, subject heading word, floating sub-heading word, keyword heading word, organism supplementary concept word, protocol supplementary concept word, rare disease supplementary concept word, unique identifier, synonyms, population supplementary concept word, anatomy supplementary concept word]
3. (primiparous or multiparous or parity or "first-time pregnan*" or "first time pregnan*").mp. [mp=title, book title, abstract, original title, name of substance word, subject heading word, floating sub-heading word, keyword heading word, organism supplementary concept word, protocol supplementary concept word, rare disease supplementary concept word, unique identifier, synonyms, population supplementary concept word, anatomy supplementary concept word]
4. 1 or 2
5. health behavior/ or health risk behaviors/
6. (folic acid or "folic acid supplement*").mp. [mp=title, book title, abstract, original title, name of substance word, subject heading word, floating sub-heading word, keyword heading word, organism supplementary concept word, protocol supplementary concept word, rare disease supplementary concept word, unique identifier, synonyms, population supplementary concept word, anatomy supplementary concept word]
7. 4 or 5
8. 3 and 6
9. limit 7 to (english language and yr="1994 -Current")
10. (cohort or "case control" or "case-control" or "cross-sectional" or "cross sectional" or "observational" or "epidemiolog*").mp. [mp=title, book title, abstract, original title, name of substance word, subject heading word, floating sub-heading word, keyword heading word, organism supplementary concept word, protocol supplementary concept word, rare disease supplementary concept word, unique identifier, synonyms, population supplementary concept word, anatomy supplementary concept word]
11. 8 and 9
12. **CINAHL Ultimate:**

Filters applied: Language – English; Date range: From 1994 to 2024

1. pregnan*
2. primiparous
3. multiparous
4. parity
5. "first-time pregnan*"
6. "first time pregnan*"
7. S2 OR S3 OR S4 OR S5 OR S6
8. health behavior
9. health risk behaviors
10. S8 OR S9
11. “folic acid”
12. “folic acid supplement*”
13. S11 OR S12
14. cohort
15. "case control"
16. "case-control"
17. "cross-sectional"
18. "cross sectional"
19. observational
20. epidemiolog*
21. S14 OR S15 OR S16 OR S17 OR S18 OR 19 OR S20
22. **SCOPUS:**

(TITLE-ABS-KEY(primiparous OR multiparous OR parity OR "first-time pregnan*" OR "first time pregnan*") AND TITLE-ABS-KEY("folic acid " OR "folic acid supplement*") AND TITLE-ABS-KEY(cohort OR "case control" OR "case-control" OR "cross-sectional" OR "cross sectional" OR observational OR epidemiolog*)) AND PUBYEAR > 1993 AND ( LIMIT-TO ( EXACTKEYWORD,"Human" ) ) AND ( LIMIT-TO ( LANGUAGE,"English" ) )

1. **Proquest Medical:**

(((primiparous OR multiparous OR parity OR "first-time pregnan*" OR "first time pregnan*") AND ("folic acid " OR "folic acid supplement*") AND noft((cohort OR "case control" OR "case-control" OR "cross-sectional" OR "cross sectional" OR observational OR epidemiolog*))) AND mainsubject(pregnancy)) AND la.exact("English") AND pd(1994-2024)

**SUPPLEMENTARY MATERIAL 2: DESCRIPTION OF STUDIES**

| **Study ID** | **Population** | | | | | **FAS Outcomes** | | | | **Review Decision** |
| --- | --- | --- | --- | --- | --- | --- | --- | --- | --- | --- |
|  | **Study design** | **Country and Study Period** | **Sample size** | **Study setting** | **Measurement tool** | **Preconception** | **Periconception** | **Postconception** | **High-risk** |  |
| Abdullahi 2014^1^ | Cross-sectional | Sudan; 1999 | 856 | Hospital-based | Questionnaire (antenatal) |  |  | X |  | Included |
| Abdulrazzaq 2003^2^ | Cross-sectional | United Arab Emirates; 2012 | 336 | Hospital-based | Questionnaire (postnatal) | X |  | X |  | Included |
| Adebo 2017^3^ | Cross-sectional | Nigeria | 300 | Hospital-based | Questionnaire (antenatal) |  |  | X |  | Included |
| Akyildiz 2024^4^ | Cross-sectional | Turkiye; 2022 | 410 | Hospital-based | Questionnaire (antenatal) |  | X |  |  | Included |
| Alsammani 2017^5^ | Cross-sectional | Sudan; 2014 | 1,000 | Hospital-based | Questionnaire (antenatal) | X |  | X |  | Included |
| Aoun 2018^6^ | Cross-sectional | Lebanon; 2013 | 465 | Hospital-based | Questionnaire (antenatal) | X |  | X |  | Included |
| Baraka 2011^7^ | Cross-sectional | Belgium; 2009 | 343 | Hospital-based | Questionnaire (antenatal) |  |  | X |  | Included |
| Barebring 2018^8^ | Cohort | Sweden; 2013-2014 | 2,109 | Population-based | Questionnaire (antenatal) |  |  | X |  | Included |
| BerinDoru 2023^9^ | Cross-sectional | Germany; 2017-2019 | 881 | Population-based | Questionnaire (postnatal) |  | X |  |  | Included |
| Bian 2021^10^ | Cross-sectional | China; 2013-2017 | 197,346 | Population-based | Medical records | X |  |  |  | Included |
| Bixenstine 2015^11^ | Cross-sectional | United States; 2009-2011 | 4,426 | Population-based | Medical records | X |  |  |  | Included |
| Bjorke-Monsen 2013^12^ | Cohort | Norway; 2002-2003 | 2,911 | Population-based | Questionnaire (antenatal) |  | X |  |  | Included |
| Bower 2005^13^ | Case-control | Australia; 1997-2000 | 578 | Population-based | Questionnaire (postnatal) |  | X |  |  | Included |
| Braekke 2003^14^ | Cross-sectional | Norway; 2001 | 1,541 | Hospital-based | Questionnaire (antenatal) |  | X |  |  | Included |
| Camier 2019^15^ | Cohort | France; 2011 | 14,156 | Population-based | Questionnaire (postnatal) |  | X | X |  | Included |
| Carmichael 2006^16^ | Case-control | United States; 1997-2000 | 2,518 | Population-based | Questionnaire (postnatal) |  | X | X |  | Included |
| Cawley 2016^17^ | Cross-sectional | Ireland | 587 | Hospital-based | Questionnaire (antenatal) | X |  |  |  | Included |
| Cawley 2017^18^ | Cross-sectional | Ireland; 2014-2016 | 856 | Hospital-based | Questionnaire (antenatal) | X |  |  |  | Included |
| Cui 2021^19^ | Cross-sectional | China; 2016 | 428 | Hospital-based | Questionnaire (antenatal) | X |  |  |  | Included |
| daRosa 2019^20^ | Cross-sectional | Brazil; 2016-2017 | 765 | Hospital-based | Questionnaire (postnatal) |  | X |  |  | Included |
| deJong-VandenBerg 2005^21^ | Case-control | United States and Canada; 1988-2002 | 7,555 | Population-based | Questionnaire (postnatal) |  | X |  |  | Included |
| DeSilva 2019^22^ | Cross-sectional | Sri Lanka | 350 | Hospital-based | Questionnaire (antenatal) | X |  |  |  | Included |
| Dessie 2017^23^ | Cross-sectional | Ethiopia; 2014 | 422 | Hospital-based | Questionnaire (antenatal) |  |  | X |  | Included |
| deWalle 2008^24^ | Cross-sectional | Netherlands; 1995-2005 | 448 | Hospital-based | Questionnaire (antenatal) |  | X |  |  | Included |
| Forster 2009^25^ | Cross-sectional | Australia | 588 | Hospital-based | Questionnaire (antenatal) |  |  | X |  | Included |
| Ha 2019^26^ | Cohort | Vietnam; 2015-2016 | 2,030 | Population-based | Questionnaire (antenatal) |  | X |  |  | Included |
| Hao 2015^27^ | Cross-sectional | China; 2013-2014 | 153 | Hospital-based | Questionnaire (postnatal) |  |  |  | X | Included |
| He 2023^28^ | Cross-sectional | China; 2016 | 12,403 | Population-based | Medical records | X |  |  |  | Included |
| Ikeda-Sakai 2019^29^ | Cross-sectional | Japan; 2005-2016 | 456 | Population-based | Medical records |  |  |  | X | Included |
| Jawad 2019^30^ | Cross-sectional | United Kingdom; 2011-2012 | 1,173 | Hospital-based | Questionnaire (antenatal) |  |  | X |  | Included |
| Jin 2023^31^ | Cross-sectional | China; 2021-2022 | 140 | Hospital-based | Questionnaire (antenatal) |  | X |  |  | Included |
| Jou 2010^32^ | Cross-sectional | Taiwan; 2008 | 275 | Hospital-based | Questionnaire (antenatal) | X |  | X |  | Included |
| Kallen 2004^33^ | Cross-sectional | Sweden | 6,953 | Population-based | Medical records |  |  | X |  | Included |
| Kamura 2022^34^ | Cross-sectional | Japan; 2017 | 1,531 | Hospital-based | Medical records |  |  | X |  | Included |
| Kikuchi 2022^35^ | Cross-sectional | Japan; 2013-2017 | 21,840 | Population-based | Both | X |  |  |  | Included |
| Kinnunen 2017^36^ | Cohort | Norway; 2008-2010 | 811 | Population-based | Questionnaire (antenatal) | X |  | X |  | Included |
| Knapik 2018^37^ | Cross-sectional | Poland; 2016-2017 | 505 | Hospital-based | Questionnaire (antenatal) |  |  | X |  | Included |
| Knudsen 2004^38^ | Cohort | Denmark; 2000-2002 | 13,680 | Population-based | Questionnaire (antenatal) |  | X |  |  | Included |
| KokuSisayTamirat 2022^39^ | Cross-sectional | Ethiopia; 2019 | 3,979 | Population-based | Medical records |  |  | X |  | Included |
| Li 2007^40^ | Case-control | China; 2003-2005 | 480 | Population-based | Questionnaire (postnatal) |  | X |  |  | Included |
| Li 2019^41^ | Cross-sectional | China; 2016-2017 | 791 | Population-based | Questionnaire (antenatal) |  | X |  |  | Included |
| Linhares 2017^42^ | Cross-sectional | Brazil; 2013 | 2,685 | Population-based | Questionnaire (postnatal) |  |  | X |  | Included |
| Liu 2019^43^ | Cross-sectional | China; 2013 | 28,678 | Population-based | Questionnaire (postnatal) |  | X |  |  | Included |
| Livock 2017^44^ | Cohort | Australia; 2011-2012 | 1,379 | Population-based | Questionnaire (antenatal) | X |  |  |  | Included |
| Lunet 2008^45^ | Case-control | Portugal; 1996 | 836 | Population-based | Questionnaire (postnatal) |  |  | X |  | Included |
| Malek 2016^46^ | Cross-sectional | Australia; 2013 | 857 | Population-based | Questionnaire (antenatal) |  | X |  |  | Included |
| Mannien 2014^47^ | Cross-sectional | Netherlands; 2009-2010 | 5,975 | Population-based | Questionnaire (antenatal) | X |  |  |  | Included |
| McGuire 2010^48^ | Cohort | Ireland; 2000-2007 | 61,252 | Hospital-based | Questionnaire (antenatal) |  | X |  |  | Included |
| McKeating 2015^49^ | Cross-sectional | Ireland; 2009-2013 | 42,042 | Hospital-based | Medical records |  | X |  |  | Included |
| Miani 2021^50^ | Cohort | Germany; 2013-2016 | 947 | Population-based | Medical records | X |  |  |  | Included |
| Miller 2011^51^ | Cross-sectional | Canada; 2006 | 6,421 | Population-based | Both | X |  |  |  | Included |
| Mohammed 2013^52^ | Cross-sectional | Ethiopia | 339 | Hospital-based | Questionnaire (antenatal) |  |  | X |  | Included |
| Mohammed 2020^53^ | Cross-sectional | Ghana; 2017 | 303 | Population-based | Questionnaire (antenatal) |  | X |  |  | Excluded |
| Morin 2002^54^ | Cross-sectional | Canada; 1999-2000 | 1,069 | Population-based | Questionnaire (antenatal) | X |  |  |  | Included |
| Morin 2002^55^ | Cross-sectional | Canada; 1999-2000 | 1,858 | Population-based | Questionnaire (antenatal) | X |  |  |  | Included |
| Mukhtar 2017^56^ | Cross-sectional | United States; 2009-2011 | 3,277 | Population-based | Both | X |  |  |  | Included |
| Navarrete-Munoz 2015^57^ | Cohort | Spain; 2003-2008 | 2,332 | Population-based | Questionnaire (antenatal) |  | X | X |  | Included |
| Nawapun 2007^58^ | Cross-sectional | Thailand; 2005 | 401 | Hospital-based | Questionnaire (antenatal) |  | X |  |  | Excluded |
| Nilsen 2006^59^ | Cohort | Norway; 2000-2003 | 22,500 | Population-based | Both |  | X |  |  | Included |
| Nilsen 2016^60^ | Cross-sectional | Italy; 2012 | 2,189 | Population-based | Questionnaire (postnatal) | X |  |  |  | Included |
| Nisar 2014^61^ | Cross-sectional | Pakistan; 2011-2012 | 6,266 | Population-based | Questionnaire (postnatal) |  |  | X |  | Included |
| Obara 2017^62^ | Cohort | Japan; 2011-2014 | 9,849 | Population-based | Both | X |  |  |  | Included |
| Ogundipe 2012^63^ | Cross-sectional | Tanzania; 1999-2008 | 21,889 | Hospital-based | Questionnaire (postnatal) |  |  | X |  | Included |
| Patti 2022^64^ | Cohort | Canada; 2008-2011 | 1,893 | Population-based | Questionnaire (antenatal) |  |  | X |  | Included |
| Pouchieu 2013^65^ | Cohort | France; 2012-2013 | 903 | Population-based | Questionnaire (antenatal) |  |  | X |  | Included |
| Ren 2006^66^ | Cross-sectional | China; 2002-2004 | 693 | Hospital-based | Questionnaire (antenatal) |  |  | X |  | Included |
| Richard-Tremblay 2012^67^ | Cross-sectional | Canada; 2010 | 361 | Hospital-based | Questionnaire (antenatal) |  | X |  |  | Included |
| Sing 2014^68^ | Cross-sectional | Nepal; 2013 | 406 | Hospital-based | Questionnaire (postnatal) |  |  | X |  | Included |
| Singh 2020^69^ | Cross-sectional | India; 2015-2016 | 190,898 | Population-based | Questionnaire (postnatal) |  |  | X |  | Included |
| Tam 2005^70^ | Cross-sectional | Canada | 383 | Hospital-based | Questionnaire (postnatal) |  | X |  |  | Included |
| Tamim 2009^71^ | Cross-sectional | Lebanon; 2003-2005 | 5,280 | Population-based | Both | X |  |  |  | Included |
| Tang 2024^72^ | Cohort | Australia; 2018-2022 | 48,045 | Population-based | Medical records |  |  | X |  | Included |
| Teixeira 2018^73^ | Cohort | New Zealand; 2009-2010 | 6,822 | Population-based | Questionnaire (antenatal) |  | X |  |  | Included |
| Timmermans 2008^74^ | Cohort | Netherlands; 2002-2006 | 6,940 | Population-based | Questionnaire (antenatal) | X |  |  |  | Included |
| Tort 2013^75^ | Cross-sectional | France; 2010 | 12,646 | Population-based | Medical records | X |  |  |  | Included |
| Tutty 2023^76^ | Cross-sectional | Australia; 2020 | 398 | Population-based | Questionnaire (postnatal) | X |  |  |  | Included |
| Wegner 2020^77^ | Cross-sectional | Germany; 2019 | 1,004 | Population-based | Questionnaire (postnatal) |  | X |  |  | Included |
| Wen 2016^78^ | Cohort | Canada; 2002-2008 | 7,669 | Population-based | Questionnaire (postnatal) |  |  | X |  | Included |
| Wojtowicz 2022^79^ | Cross-sectional | Poland; 2017-2019 | 1,455 | Hospital-based | Medical records |  |  |  | X | Included |
| Xing 2012^80^ | Cohort | China; 2008-2009 | 4,290 | Population-based | Questionnaire (antenatal) |  | X |  |  | Included |
| Yamamoto 2018^81^ | Cross-sectional | Japan; 2014-2015 | 2,012 | Hospital-based | Questionnaire (antenatal) |  | X |  |  | Included |
| Yan 2017^82^ | Cross-sectional | China; 2015-2016 | 1,921 | Hospital-based | Questionnaire (postnatal) |  | X |  |  | Included |
| Yang 2022^83^ | Cross-sectional | China; 2021 | 821 | Population-based | Questionnaire (antenatal) |  | X |  |  | Included |

**References**

1. Abdullahi H, Gasim GI, Saeed A, Imam AM, Adam I. Antenatal iron and folic acid supplementation use by pregnant women in Khartoum, Sudan. BMC Res Notes. 2014 Aug 7;7(1):498.

2. Abdulrazzaq YM, Al-Gazali LI, Bener A, Hossein M, Verghese M, Dawodu A, et al. Folic acid awareness and intake survey in the United Arab Emirates. Reprod Toxicol. 2003;17(2):171–6.

3. Adebo OO, Dairo DM, Ndikom CM, Adejumo PO. Knowledge and uptake of folic acid among pregnant women attending a secondary health facility in Nigeria. Br J Midwifery. 2017;25(6):358–64.

4. Akyıldız D, Alkan H. Factors Associated with the use of Folic Acid and Iron Supplementation in the Periconceptional and Antenatal Periods of Pregnant Women: A Cross-Sectional Study. J Acad Res Nurs JAREN. 2024;10(1):9–20.

5. Alsammani MA, Kunna A, Adam EM. Factors associated with folic acid knowledge and intake among pregnant women in Sudan. East Mediterr Health J Rev Sante Mediterr Orient Al-Majallah Al-Sihhiyah Li-Sharq Al-Mutawassit. 2017;23(10):662–9.

6. Aoun A, Faddoul L, El Jabbour F, El Osta N, Hlais S, El Osta L. Are the Level of Knowledge and Practices of Pregnant Women Regarding Folic Acid Supplementation Still Inadequate? A Cross-Sectional Study in a Middle Eastern Urban Setting. J Diet Suppl. 2018;15(5):692–703.

7. Baraka MA, Steurbaut S, Leemans L, Foulon W, Laubach M, Coomans D, et al. Determinants of folic acid use in a multi-ethnic population of pregnant women: a cross-sectional study. J Perinat Med. 2011 Nov 1;39(6):685–92.

8. Bärebring L, Mullally D, Glantz A, Elllis J, Hulthén L, Jagner A, et al. Sociodemographic factors associated with dietary supplement use in early pregnancy in a Swedish cohort. Br J Nutr. 2018;119(1):90–5.

9. Berin Doru, Hockamp N, Sievers E, Hülk P, Lücke T, Kersting M. Adherence to recommendations for nutrient supplementation related to pregnancy in Germany. Food Sci Nutr. 2023;11(9):5236–47.

10. Bian H, Tang Y, Zhou Y, Li H, Liu J. Demographic variations and temporal trends in prenatal use of multiple micronutrient supplements in Beijing, 2013–2017. Public Health Nutr. 2021;24(5):826–33.

11. Bixenstine PJ, Cheng TL, Cheng D, Connor KA, Mistry KB. Association Between Preconception Counseling and Folic Acid Supplementation Before Pregnancy and Reasons for Non-Use. Matern Child Health J. 2015 Sept 1;19(9):1974–84.

12. Bjorke-Monsen AL, Roth C, Magnus P, Midttun O, Nilsen RM, Reichborn-Kjennerud T, et al. Maternal B vitamin status in pregnancy week 18 according to reported use of folic acid supplements. Mol Nutr Food Res. 2013;57(4):645–52.

13. Bower C, Miller M, Payne J, Serna P. Promotion of folate for the prevention of neural tube defects: who benefits?. Paediatr Perinat Epidemiol. 2005;19(6):435–44.

14. Braekke K, Staff AC. Periconceptional use of folic acid supplements in Oslo. Acta Obstet Gynecol Scand. 2003;82(7):620–7.

15. Camier A, Kadawathagedara M, Lioret S, Bois C, Cheminat M, Dufourg MN, et al. Social Inequalities in Prenatal Folic Acid Supplementation: Results from the ELFE Cohort. Nutrients. 2019;11(5).

16. Carmichael SL, Shaw GM, Yang W, Laurent C, Herring A, Royle MH, et al. Correlates of intake of folic acid-containing supplements among pregnant women. Am J Obstet Gynecol. 2006;194(1):203–10.

17. Cawley S, Mullaney L, McKeating A, Farren M, McCartney D, Turner MJ. An analysis of folic acid supplementation in women presenting for antenatal care. J Public Health. 2016;38(1):122–9.

18. Cawley S, Mullaney L, Kennedy R, Farren M, McCartney D, Turner MJ. Duration of periconceptional folic acid supplementation in women booking for antenatal care. Public Health Nutr. 2017;20(2):371–9.

19. Cui M, Lu XL, Lyu YY, Wang F, Xie XL, Cheng XY, et al. Knowledge and intake of folic acid to prevent neural tube defects among pregnant women in urban China: a cross-sectional study. BMC Pregnancy Childbirth. 2021;21(1):432.

20. da Rosa EB, Silveira DB, Correia JD, Grapiglia CG, de Moraes SAG, Nunes MR, et al. Periconceptional folic acid supplementation in Southern Brazil: Why are not we doing it right? Am J Med Genet Part A. 2019;179(1):20–8.

21. de Jong-Van den Berg LTW, Hernandez-Diaz S, Werler MM, Louik C, Mitchell AA. Trends and predictors of folic acid awareness and periconceptional use in pregnant women. Am J Obstet Gynecol. 2005;192(1):121–8.

22. De Silva J, Amarasena S, Jayaratne K, Perera B. Correlates of knowledge on birth defects and associated factors among antenatal mothers in Galle, Sri Lanka: A cross-sectional analytical study. BMC Pregnancy Childbirth [Internet]. 2019;19(1). Available from: https://www.scopus.com/inward/record.uri?eid=2-s2.0-85060134005&doi=10.1186%2fs12884-018-2163-9&partnerID=40&md5=c0af41afa5b3e613dd39cafa8bc24aeb

23. Dessie MA, Zeleke EG, Workie SB, Berihun AW. Folic acid usage and associated factors in the prevention of neural tube defects among pregnant women in Ethiopia: cross-sectional study. BMC Pregnancy Childbirth. 2017;17(1):313.

24. de Walle HEK, de Jong-van den Berg LTW. Ten years after the Dutch public health campaign on folic acid: the continuing challenge. Eur J Clin Pharmacol. 2008;64(5):539–43.

25. Forster DA, Wills G, Denning A, Bolger M. The use of folic acid and other vitamins before and during pregnancy in a group of women in Melbourne, Australia. Midwifery. 2009;25(2):134–46.

26. Ha AVV, Zhao Y, Binns CW, Pham NM, Nguyen CL, Nguyen PTH, et al. Low Prevalence of Folic Acid Supplementation during Pregnancy: A Multicenter Study in Vietnam. Nutrients. 2019;11(10):2347–2347.

27. Hao N, Xia W, Tang Y, Wu M, Jiang H, Lin X, et al. Periconceptional folic acid supplementation among pregnant women with epilepsy in a developing country: a retroprospective survey in China. Epilepsy Behav EB. 2015;44(100892858):27–34.

28. He G, Yang H, Chen M, Liu X. Factors influencing folic acid, multivitamin, and calcium supplementation among pregnant women in China based on a national cross-sectional survey. Chin Med J (Engl). 2023;136(4):473–5.

29. Ikeda-Sakai Y, Saito Y, Obara T, Goto M, Sengoku T, Takahashi Y, et al. Inadequate Folic Acid Intake Among Women Taking Antiepileptic Drugs During Pregnancy in Japan: A Cross-Sectional Study. Sci Rep. 2019 Sept 18;9(1):13497.

30. Jawad A, Patel D, Brima N, Stephenson J. Alcohol, smoking, folic acid and multivitamin use among women attending maternity care in London: A cross-sectional study. Sex Reprod Healthc. 2019 Dec 1;22:100461.

31. Jin YJ, Kim HW. Influence of folic acid knowledge on effective folic acid intake in Chinese pregnant women: a cross-sectional study. Korean J Women Health Nurs. 2023;29(4):291–301.

32. Jou HJ, Hsu IP, Liu CY, Chung SH, Chen SM, Gau ML. Awareness and use of folic acid among pregnant women in Taipei. Taiwan J Obstet Gynecol. 2010;49(3):306–10.

33. Källén B. Use of folic acid supplementation and risk for dizygotic twinning. Early Hum Dev. 2004;80(2):143–51.

34. Kamura S, Sasaki A, Ogawa K, Kato K, Sago H. Periconceptional folic acid intake and disturbing factors: A single-center study in Japan. Congenit Anom. 2022;62(1):42–6.

35. Kikuchi D, Obara T, Usuzaki T, Yonezawa Y, Yamashita T, Oyanagi G, et al. Evaluating folic acid supplementation among Japanese pregnant women with dietary intake of folic acid lower than 480 microg per day: results from TMM BirThree Cohort Study. J Matern-Fetal Neonatal Med Off J Eur Assoc Perinat Med Fed Asia Ocean Perinat Soc Int Soc Perinat Obstet. 2022;35(5):964–9.

36. Kinnunen TI, Sletner L, Sommer C, Post MC, Jenum AK. Ethnic differences in folic acid supplement use in a population-based cohort of pregnant women in Norway. BMC Pregnancy Childbirth. 2017 May 15;17(1):143.

37. Knapik A, Kocot K, Witek A, Jankowski M, Wroblewska-Czech A, Kowalska M, et al. Dietary supplementation usage by pregnant women in Silesia - population based study. Ginekol Pol. 2018;89(9):506–12.

38. Knudsen VK, Orozova-Bekkevold I, Rasmussen LB, Mikkelsen TB, Michaelsen KF, Olsen SF. Low compliance with recommendations on folic acid use in relation to pregnancy: is there a need for fortification?. Public Health Nutr. 2004;7(7):843–50.

39. Koku Sisay Tamirat, Kebede FB, Gonete TZ, Tessema GA, Zemenu Tadesse Tessema. Geographical variations and determinants of iron and folic acid supplementation during pregnancy in Ethiopia: analysis of 2019 mini demographic and health survey. BMC Pregnancy Childbirth. 2022;22:1–11.

40. Li Z, Ren A, Zhang L, Liu J. Periconceptional use of folic acid in Shanxi Province of northern China. Public Health Nutr. 2007;10(5):471–6.

41. Li D, Huang L, Yang W, Qi C, Shang L, Xin J, et al. Knowledge, attitude and practice level of women at the periconceptional period: a cross-sectional study in Shaanxi China. BMC Pregnancy Childbirth. 2019;19(1):326.

42. Linhares AO, Juraci Almeida Cesar. Folic acid supplementation among pregnant women in southern Brazil: prevalence and factors associated. Ciênc Saúde Coletiva [Internet]. 2017;22(2). Available from: https://www.proquest.com/scholarly-journals/folic-acid-supplementation-among-pregnant-women/docview/1875901719/se-2?accountid=14775

43. Liu D, Cheng Y, Dang S, Wang D, Zhao Y, Li C, et al. Maternal adherence to micronutrient supplementation before and during pregnancy in Northwest China: a large-scale population-based cross-sectional survey. BMJ Open. 2019;9(8):e028843.

44. Livock M, Anderson PJ, Lewis S, Bowden S, Muggli E, Halliday J. Maternal micronutrient consumption periconceptionally and during pregnancy: a prospective cohort study. Public Health Nutr. 2017;20(2):294–304.

45. Lunet N, Rodrigues T, Correia S, Barros H. Adequacy of prenatal care as a major determinant of folic acid, iron, and vitamin intake during pregnancy. Cad Saude Publica. 2008;24(5):1151–7.

46. Malek L, Umberger W, Makrides M, Zhou SJ. Poor adherence to folic acid and iodine supplement recommendations in preconception and pregnancy: a cross-sectional analysis. Aust N Z J Public Health. 2016;40(5):424–9.

47. Mannien J, de Jonge A, Cornel MC, Spelten E, Hutton EK. Factors associated with not using folic acid supplements preconceptionally. Public Health Nutr. 2014;17(10):2344–50.

48. McGuire M, Cleary B, Sahm L, Murphy DJ. Prevalence and predictors of periconceptional folic acid uptake—prospective cohort study in an Irish urban obstetric population. Hum Reprod. 2010 Feb 1;25(2):535–43.

49. McKeating A, Farren M, Cawley S, Daly N, McCartney D, Turner MJ. Maternal folic acid supplementation trends 2009-2013. Acta Obstet Gynecol Scand. 2015;94(7):727–33.

50. Miani C, Ludwig A, Doyle IM, Breckenkamp J, Hoeller-Holtrichter C, Spallek J, et al. The role of education and migration background in explaining differences in folic acid supplementation intake in pregnancy: results from a German birth cohort study. Public Health Nutr. 2021;24(18):6094–102.

51. Miller EC, Liu N, Wen SW, Walker M. Why do Canadian women fail to achieve optimal pre-conceptional folic acid supplementation? An observational study. J Obstet Gynaecol Can JOGC J Obstet Gynecol Can JOGC. 2011;33(11):1116–23.

52. Mohammed MA BPharm, MSc(clinpharm), Bushra AW BSc, MPHE, Aljadhey HS PharmD, PhD, Ahmed JH BPharm, MSc. Supplement Use Among Pregnant Women in Ethiopia: Prevalence and Predictors. Ther Innov Regul Sci. 2013;47(4):416–23.

53. Mohammed BS, Kawawa AR, Wemakor A. Prevalence and determinants of uptake of folic acid in peri-conceptional period in a rural lower-middle-income country, Ghana. Basic Clin Pharmacol Toxicol. 2020;126(3):254–62.

54. Morin P, De Wals P, St-Cyr-Tribble D, Niyonsenga T, Payette H, Morin P, et al. Pregnancy planning: a determinant of folic acid supplements use for the primary prevention of neural tube defects. Can J Public Health. 2002;93(4):259–63.

55. Morin P, De Wals P, Noiseux M, Niyonsenga T, St-Cyr-Tribble D, Tremblay C. Pregnancy planning and folic acid supplement use: results from a survey in Quebec. Prev Med. 2002;35(2):143–9.

56. Mukhtar A, Kramer MR, Oakley Jr GP, Kancherla V. Race and ethnicity and preconception folic acid supplement use among pregnant women in Georgia, PRAMS 2009 to 2011. Birth Defects Res. 2017;109(1):38–48.

57. Navarrete-Muñoz EM, Valera-Gran D, De La Hera MG, Gimenez-Monzo D, Morales E, Julvez J, et al. Use of high doses of folic acid supplements in pregnant women in Spain: An INMA cohort study. BMJ Open [Internet]. 2015;5(11). Available from: https://www.scopus.com/inward/record.uri?eid=2-s2.0-85006817994&doi=10.1136%2fbmjopen-2015-009202&partnerID=40&md5=e582d8924ab237e77999abf340b4b4a2

58. Nawapun K, Phupong V. Awareness of the benefits of folic acid and prevalence of the use of folic acid supplements to prevent neural tube defects among Thai women. Arch Gynecol Obstet. 2007;276(1):53–7.

59. Nilsen RM, Vollset SE, Gjessing HK, Magnus P, Meltzer HM, Haugen M, et al. Patterns and predictors of folic acid supplement use among pregnant women: the Norwegian Mother and Child Cohort Study. Am J Clin Nutr. 2006 Nov 1;84(5):1134–41.

60. Nilsen RM, Leoncini E, Gastaldi P, Allegri V, Agostino R, Faravelli F, et al. Prevalence and determinants of preconception folic acid use: an Italian multicenter survey. Ital J Pediatr. 2016 July 13;42(1):65.

61. Nisar YB, Dibley MJ, Mir AM. Factors associated with non-use of antenatal iron and folic acid supplements among Pakistani women: a cross sectional household survey. BMC Pregnancy Childbirth. 2014;14(100967799):305.

62. Obara T, Nishigori H, Nishigori T, Metoki H, Ishikuro M, Tatsuta N, et al. Prevalence and determinants of inadequate use of folic acid supplementation in Japanese pregnant women: the Japan Environment and Children’s Study (JECS). J Matern-Fetal Neonatal Med Off J Eur Assoc Perinat Med Fed Asia Ocean Perinat Soc Int Soc Perinat Obstet. 2017;30(5):588–93.

63. Ogundipe O, Hoyo C, Stbye T, Oneko O, Manongi R, Lie RT, et al. Factors associated with prenatal folic acid and iron supplementation among 21,889 pregnant women in Northern Tanzania: A cross-sectional hospital-based study. BMC Public Health [Internet]. 2012;12(1). Available from: https://www.scopus.com/inward/record.uri?eid=2-s2.0-84862683322&doi=10.1186%2f1471-2458-12-481&partnerID=40&md5=fe31f4f2734299dc5f4ff2e7f5a565bb

64. Patti MA, Braun JM, Arbuckle TE, MacFarlane AJ. Associations between folic acid supplement use and folate status biomarkers in the first and third trimesters of pregnancy in the Maternal-Infant Research on Environmental Chemicals (MIREC) Pregnancy Cohort Study. Am J Clin Nutr. 2022;116(6):1852–63.

65. Pouchieu C, Lévy R, Faure C, Andreeva VA, Galan P, Hercberg S, et al. Socioeconomic, Lifestyle and Dietary Factors Associated with Dietary Supplement Use during Pregnancy. PLoS ONE [Internet]. 2013;8(8). Available from: https://www.scopus.com/inward/record.uri?eid=2-s2.0-84881531635&doi=10.1371%2fjournal.pone.0070733&partnerID=40&md5=0c0e36301bd54202005343e1bf5cc078

66. Ren A, Zhang L, Li Z, Hao L, Tian Y, Li Z. Awareness and use of folic acid, and blood folate concentrations among pregnant women in northern China--an area with a high prevalence of neural tube defects. Reprod Toxicol Elmsford N. 2006;22(3):431–6.

67. Richard-Tremblay AA, Sheehy O, Audibert F, Ferreira E, Berard A. Concordance between periconceptional folic acid supplementation and Canadian Clinical Guidelines. J Popul Ther Clin Pharmacol J Ther Popul Pharmacol Clin. 2012;19(2):e150-9.

68. Sing SR, Ratanasiri T, Thapa P, Koju R, Ratanasiri A, Arkaravichien T, et al. Effect of knowledge and perception on adherence to iron and folate supplementation during pregnancy in Kathmandu, Nepal. J Med Assoc Thail Chotmaihet Thangphaet. 2014;97 Suppl 10(izr, 7507216):S67-74.

69. Singh PK, Dubey R, Singh L, Kumar C, Rai RK, Singh S. Public health interventions to improve maternal nutrition during pregnancy: A nationally representative study of iron and folic acid consumption and food supplements in India. Public Health Nutr. 2020;23(15):2671–86.

70. Tam LE, McDonald SD, Wen SW, Smith GN, Windrim RC, Walker MC. A survey of preconceptional folic acid use in a group of Canadian women. J Obstet Gynaecol Can JOGC J Obstet Gynecol Can JOGC. 2005;27(3):232–6.

71. Tamim H, Harrison G, Atoui M, Mumtaz G, El-Kak F, Seoud M, et al. Preconceptional folic acid supplement use in Lebanon. Public Health Nutr. 2009;12(5):687–92.

72. Tang HY, Elhindi J, Blumenthal C, Pasupathy D, Melov SJ. Are migrants during the periconception period less likely to be supplementing with folic acid: An Australian cohort study. Midwifery. 2024;132(8510930, mwf):103984.

73. Teixeira JA, Castro TG, Wall CR, Marchioni DM, Berry S, Morton SM, et al. Determinants of folic acid supplement use outside national recommendations for pregnant women: results from the Growing Up in New Zealand cohort study. Public Health Nutr. 2018;21(12):2183–92.

74. Timmermans S, Jaddoe VWV, Mackenbach JP, Hofman A, Steegers-Theunissen RPM, Steegers EAP. Determinants of folic acid use in early pregnancy in a multi-ethnic urban population in The Netherlands: The Generation R study. Prev Med. 2008 Oct 1;47(4):427–32.

75. Tort J, Lelong N, Prunet C, Khoshnood B, Blondel B. Maternal and health care determinants of preconceptional use of folic acid supplementation in France: results from the 2010 National Perinatal Survey. BJOG Int J Obstet Gynaecol. 2013;120(13):1661–7.

76. Tutty E, Wimsett J, Oyston C, Tutty S, Harwood M, Legget E, et al. Inequities in pre-pregnancy folic acid use in Central and South Auckland: secondary analysis from a postpartum contraception survey. J Prim Health Care. 2023;15(4):308–15.

77. Wegner C, Kancherla V, Lux A, Köhn A, Bretschneider D, Freese K, et al. Periconceptional folic acid supplement use among women of reproductive age and its determinants in central rural Germany: Results from a cross sectional study. Birth Defects Res. 2020;112(14):1057–66.

78. Wen SW, Guo Y, Rodger M, White RR, Yang Q, Smith GN, et al. Folic Acid Supplementation in Pregnancy and the Risk of Pre-Eclampsia-A Cohort Study. PloS One. 2016;11(2):e0149818.

79. Wojtowicz A, Babczyk D, Galas A, Skalska-Swistek M, Gorecka M, Witkowski R, et al. Evaluation of the prevalence of folic acid supplementation before conception and through the first 12 weeks of pregnancy in Polish women at high risk of fetal anomalies. Ginekol Pol. 2022;93(6):489–95.

80. Xing XY, Tao FB, Hao JH, Huang K, Huang ZH, Zhu XM, et al. Periconceptional folic acid supplementation among women attending antenatal clinic in Anhui, China: data from a population-based cohort study. Midwifery. 2012;28(3):291–7.

81. Yamamoto S, Wada Y. Awareness, use and information sources of folic acid supplementation to prevent neural tube defects in pregnant Japanese women. Public Health Nutr. 2018;21(4):732–9.

82. Yan J, Zheng YZ, Cao LJ, Liu YY, Li W, Huang GW. Periconceptional Folic Acid Supplementation in Chinese Women: A Cross-sectional Study. Biomed Environ Sci BES. 2017;30(10):737–48.

83. Yang J, Reheman Z, Liu Y, Wang Y, Wang N, Ye J, et al. The compliance of free folic acid supplements among pregnant women in rural areas of Northwestern China: The role of related knowledge. Front Public Health. 2022;10(101616579):1079779.

**SUPPLEMENTARY MATERIAL 3: ADAPTED NEWCASTLE-OTTAWA SCALE (NOS)**

**Selection of parity groups – 4 possible points:**

1. Representativeness of sample:

- Truly representative of the pregnant women in the target population (all subjects or random sampling) – 1 point
- Somewhat representative of the pregnant women in the target population (non-random sampling) – 1 point
- Pregnant women attending a particular maternity hospital – 1 point
- Selected group of pregnant women (e.g. pregnant women taking specific medication, pregnant women with a medical condition, etc.) – 0 point
- No description of the sampling strategy – 0 point

1. Inclusion and exclusion criteria

- Clearly stated – 1 point
- Not stated – 0 point

1. Recall bias

- Data was collected at booking – 1 point
- Data was collected sometime during pregnancy – 1 point
- Data was collected postpartum (before hospital discharge) – 1 point
- Data was collected postpartum (after hospital discharge) – 0 point

1. Selective reporting bias

- Reported data correspond with initial sample size – 1 point
- Reported data do not correspond with the initial sample size, but a rationale provided – 1 point
- Reported data do not correspond with the initial sample size, no rationale provided – 0 point

**Comparability of parity groups (controlling for important confounders) – 2 possible points:**

- Age – 1 point
- Pregnancy planning – 1 point
- Both – 2 points
- Neither – 0 point

**Ascertainment of outcome (FAS) – 3 possible points:**

1. Measurement of outcome

- Folic acid supplement use was defined with the timing of intake – 1 point
- Definition does not specify the timing of FAS – 0 point
- No definition for folic acid intake – 0 point

1. Validity of measurement tool

- Questions about folic acid supplementation are described – 1 point
- No description of the measurement tool or questions about FAS – 0 point

1. Statistical test

- The statistical test used to analyse FAS vs parity is clearly described and appropriate, and the measurement of the association is presented, including confidence intervals – 1 point
- The statistical test is not appropriate, not described or incomplete (does not include measures of effect and confidence intervals) – 0 point

**SUPPLEMENTARY MATERIAL 4: RISK OF BIAS ASSESSMENT OF INCLUDED STUDIES**

| **Study ID** | **Selection of parity groups** | **Comparability of parity groups** | **Ascertainment of folic acid intake** | **Overall risk of bias** |
| --- | --- | --- | --- | --- |
| Abdullahi 2014 | 4 | 1 | 1 | Moderate Quality |
| Abdulrazzaq 2003 | 4 | 1 | 1 | Moderate Quality |
| Adebo 2017 | 4 | 0 | 0 | Moderate Quality |
| Akyildiz 2024 | 4 | 1 | 3 | High Quality |
| Alsammani 2017 | 4 | 1 | 1 | Moderate Quality |
| Aoun 2018 | 4 | 0 | 1 | Moderate Quality |
| Baraka 2011 | 3 | 0 | 2 | Moderate Quality |
| Barebring 2018 | 4 | 1 | 2 | High Quality |
| BerinDoru 2023 | 3 | 0 | 3 | Moderate Quality |
| Bian 2021 | 4 | 1 | 3 | High Quality |
| Bixenstine 2015 | 3 | 2 | 3 | High Quality |
| Bjorke-Monsen 2013 | 3 | 0 | 1 | Moderate Quality |
| Bower 2005 | 3 | 2 | 2 | High Quality |
| Braekke 2003 | 3 | 1 | 3 | High Quality |
| Camier 2019 | 4 | 0 | 3 | High Quality |
| Carmichael 2006 | 2 | 1 | 3 | Moderate Quality |
| Cawley 2016 | 4 | 2 | 2 | High Quality |
| Cawley 2017 | 4 | 2 | 2 | High Quality |
| Cui 2021 | 4 | 0 | 2 | Moderate Quality |
| daRosa 2019 | 4 | 1 | 2 | High Quality |
| deJong-VandenBerg 2005 | 3 | 2 | 2 | High Quality |
| DeSilva 2019 | 4 | 1 | 2 | High Quality |
| Dessie 2017 | 4 | 0 | 2 | Moderate Quality |
| deWalle 2008 | 4 | 0 | 0 | Moderate Quality |
| Forster 2009 | 4 | 0 | 1 | Moderate Quality |
| Ha 2019 | 4 | 2 | 3 | High Quality |
| Hao 2015 | 2 | 2 | 3 | High Quality |
| He 2023 | 4 | 0 | 1 | Moderate Quality |
| Ikeda-Sakai 2019 | 3 | 2 | 3 | High Quality |
| Jawad 2019 | 3 | 2 | 2 | High Quality |
| Jin 2023 | 4 | 0 | 3 | High Quality |
| Jou 2010 | 3 | 1 | 2 | Moderate Quality |
| Kallen 2004 | 4 | 0 | 1 | Moderate Quality |
| Kamura 2022 | 4 | 1 | 1 | Moderate Quality |
| Kikuchi 2022 | 4 | 1 | 2 | High Quality |
| Kinnunen 2017 | 4 | 2 | 3 | High Quality |
| Knapik 2018 | 2 | 0 | 0 | Low Quality |
| Knudsen 2004 | 4 | 1 | 3 | High Quality |
| KokuSisayTamirat 2022 | 3 | 1 | 3 | High Quality |
| Li 2007 | 4 | 0 | 1 | Moderate Quality |
| Li 2019 | 4 | 1 | 3 | High Quality |
| Linhares 2017 | 4 | 1 | 2 | High Quality |
| Liu 2019 | 3 | 1 | 3 | High Quality |
| Livock 2017 | 4 | 2 | 2 | High Quality |
| Lunet 2008 | 4 | 1 | 1 | Moderate Quality |
| Malek 2016 | 4 | 0 | 3 | High Quality |
| Mannien 2014 | 4 | 1 | 3 | High Quality |
| McGuire 2010 | 4 | 2 | 3 | High Quality |
| McKeating 2015 | 4 | 2 | 3 | High Quality |
| Miani 2021 | 1 | 2 | 3 | Moderate Quality |
| Miller 2011 | 2 | 1 | 3 | Moderate Quality |
| Mohammed 2013 | 4 | 0 | 1 | Moderate Quality |
| Mohammed 2020 | 3 | 0 | 0 | Low Quality |
| Morin 2002 | 4 | 2 | 3 | High Quality |
| Morin 2002 | 4 | 2 | 3 | High Quality |
| Mukhtar 2017 | 3 | 2 | 3 | High Quality |
| Navarrete-Munoz 2015 | 4 | 2 | 3 | High Quality |
| Nawapun 2007 | 4 | 0 | 2 | Moderate Quality |
| Nilsen 2006 | 4 | 1 | 3 | High Quality |
| Nilsen 2016 | 4 | 2 | 2 | High Quality |
| Nisar 2014 | 3 | 0 | 3 | Moderate Quality |
| Obara 2017 | 4 | 1 | 2 | High Quality |
| Ogundipe 2012 | 4 | 1 | 2 | High Quality |
| Patti 2022 | 4 | 0 | 1 | Moderate Quality |
| Pouchieu 2013 | 4 | 1 | 2 | High Quality |
| Ren 2006 | 4 | 1 | 2 | High Quality |
| Richard-Tremblay 2012 | 4 | 2 | 3 | High Quality |
| Sing 2014 | 3 | 0 | 1 | Moderate Quality |
| Singh 2020 | 3 | 1 | 2 | Moderate Quality |
| Tam 2005 | 4 | 0 | 2 | Moderate Quality |
| Tamim 2009 | 4 | 1 | 2 | High Quality |
| Tang 2024 | 4 | 1 | 3 | High Quality |
| Teixeira 2018 | 4 | 2 | 3 | High Quality |
| Timmermans 2008 | 4 | 2 | 3 | High Quality |
| Tort 2013 | 4 | 2 | 3 | High Quality |
| Tutty 2023 | 4 | 1 | 2 | High Quality |
| Wegner 2020 | 4 | 2 | 3 | High Quality |
| Wen 2016 | 3 | 0 | 1 | Moderate Quality |
| Wojtowicz 2022 | 3 | 1 | 2 | Moderate Quality |
| Xing 2012 | 4 | 2 | 3 | High Quality |
| Yamamoto 2018 | 4 | 1 | 2 | High Quality |
| Yan 2017 | 3 | 1 | 3 | High Quality |
| Yang 2022 | 4 | 2 | 3 | High Quality |

**SUPPLEMENTARY MATERIAL 5: SUB-GROUP ANALYSIS FOR PRECONCEPTIONAL SUPPLEMENTATION**

| **Study characteristics** | **Sub-groups** | **Number of studies** | **Odds Ratio (95% CI)** | **Between-group difference (p-value)** | **Cochrane’s Q statistic (p-value)** | **I^2^ statistic** |
| --- | --- | --- | --- | --- | --- | --- |
| Overall (preconception) | - | 25 | 0.710 (0.642 – 0.785) | - | 211.75 (p < 0.001) | 88.67% |
| Study design | Cohort | 5 | 0.696 (0.561 – 0.864) | 0.04 (0.840) | 14.61 (0.006) | 72.62% |
|  | Cross-sectional | 20 | 0.713 (0.637 – 0.799) |  | 187.18 (p < 0.001) | 89.85% |
| Study setting | Hospital-based | 7 | 0.700 (0.505 – 0.971) | 0.00 (0.951) | 16.33 (0.012) | 63.26% |
|  | Population-based | 18 | 0.708 (0.635 – 0.789) |  | 195.41 (p < 0.001) | 91.30% |
| Population | Pregnant women | 16 | 0.693 (0.624 – 0.770) | 9.29 (0.010) | 70.77 (p < 0.001) | 78.80% |
|  | Postpartum women | 7 | 0.693 (0.528 – 0.910) |  | 55.65 (p < 0.001) | 89.22% |
|  | Both | 2 | 0.857 (0.779 – 0.943) |  | 0.06 (0.800) | 0.00% |
| Method of assessing folic acid intake | Medical records | 4 | 0.666 (0.566 – 0.785) | 1.97 (0.374) | 18.76 (p < 0.001) | 84.01% |
|  | Questionnaire | 16 | 0.760 (0.674 – 0.858) |  | 42.78 (p < 0.001) | 64.94% |
|  | Both | 5 | 0.654 (0.469 – 0.912) |  | 126.80 (p < 0.001) | 96.85% |
| Definition (of preconception) adopted by studies | Before conception | 17 | 0.685 (0.613 – 0.764) | 3.92 (0.270) | 93.77 (p < 0.001) | 82.94% |
|  | 1 month before conception | 2 | 0.703 (0.580 – 0.851) |  | 1.17 (0.278) | 14.89% |
|  | 3 months before conception | 5 | 0.775 (0.631 – 0.952) |  | 23.76 (p < 0.001) | 83.16% |
|  | 3 or 1 month before conception | 1 | 1.000 (0.671 – 1.491) |  | - | - |
| Study locations by world regions | Africa | 1 | 0.950 (0.721 – 1.252) | 9.80 (0.081) | - | - |
|  | Asia | 7 | 0.649 (0.551 – 0.765) |  | 44.93 (p < 0.001) | 86.65% |
|  | Australia | 2 | 0.825 (0.562 – 1.210) |  | 1.50 (0.221) | 33.24% |
|  | Europe | 8 | 0.640 (0.533 – 0.768) |  | 41.06 (p < 0.001) | 82.95% |
|  | Middle East | 2 | 0.744 (0.546 – 1.015) |  | 0.31 (0.580) | 0.00% |
|  | North America | 4 | 0.833 (0.695 – 0.975) |  | 14.80 (0.005) | 72.98% |
| Food fortification policies | Mandatory | 6 | 0.793 (0.672 – 0.936) | 5.02 (0.170) | 9.68 (0.085) | 48.36% |
|  | Voluntary | 3 | 0.812 (0.665 – 0.993) |  | 38.14 (p < 0.001) | 94.76% |
|  | None | 5 | 0.651 (0.485 – 0.874) |  | 16.14 (0.003) | 75.22% |
|  | Not reported | 11 | 0.657 (0.576 – 0.749) |  | 41.05 (p < 0.001) | 75.64% |

**SUPPLEMENTARY MATERIAL 6: SUB-GROUP ANALYSIS FOR PERICONCEPTIONAL SUPPLEMENTATION**

| **Study characteristics** | **Sub-groups** | **Number of studies** | **Odds Ratio (95% CI)** | **Between-group difference (p-value)** | **Cochrane’s Q statistic (p-value)** | **I^2^ statistic** |
| --- | --- | --- | --- | --- | --- | --- |
| Overall | - | 29 | 0.693 (0.636 – 0.755) | - | 194.61 (p < 0.001) | 85.61% |
| Study design | Case-control | 4 | 0.875 (0.623 – 1.227) | 5.88 (0.05) | 7.20 (0.066) | 58.33% |
|  | Cohort | 9 | 0.742 (0.658 – 0.837) |  | 85.45 (p < 0.001) | 90.64% |
|  | Cross-sectional | 16 | 0.618 (0.540 – 0.707) |  | 59.41 (p < 0.001) | 74.75% |
| Study setting | Hospital-based | 11 | 0.611 (0.529 – 0.707) | 4.16 (0.041) | 67.42 (p < 0.001) | 85.17% |
|  | Population-based | 18 | 0.742 (0.661 – 0.833) |  | 121.35 (p < 0.001) | 85.99% |
| Population | Pregnant women | 18 | 0.719 (0.651 – 0.794) | 6.85 (0.033) | 74.49 (p < 0.001) | 77.18% |
|  | Postpartum women | 10 | 0.679 (0.584 – 0.791) |  | 80.74 (p < 0.001) | 88.85% |
|  | Both | 1 | 0.456 (0.328 – 0.632) |  | - | - |
| Method of assessing folic acid intake | Medical records | 3 | 0.690 (0.583 – 0.818) | 1.44 (0.487) | 37.32 (p < 0.001) | 94.64% |
|  | Questionnaire | 25 | 0.683 (0.601 – 0.777) |  | 150.72 (p < 0.001) | 84.08% |
|  | Both | 1 | 0.737 (0.695 – 0.782) |  | - | - |
| Definition (of periconception) adopted by studies | 1 month before to 1 month after conception | 1 | 0.620 (0.451 – 0.852) | 57.21(p < 0.001) | - | - |
|  | 1 month before to 2 months after conception | 3 | 0.746 (0.681 – 0.818) |  | 1.66 (0.437) | 0.00% |
|  | 1 month before to 3 months after conception | 8 | 0.757 (0.646 – 0.887) |  | 26.55 (p < 0.001) | 73.64% |
|  | 1 month before to 4 months after conception | 1 | 0.590 (0.499 – 0.698) |  | - | - |
|  | 1 month before and continued after conception | 1 | 0.465 (0.349 – 0.619) |  | - | - |
|  | 2 months before to 1 month after conception | 1 | 1.050 (0.377 – 2.926) |  | - | - |
|  | 2 months before to 3 months after conception | 2 | 0.887 (0.782 – 1.007) |  | 0.05 (0.819) | 0.00% |
|  | 3 months before to 1 month after conception | 1 | 0.611 (0.424 – 0.881) |  | - | - |
|  | 3 months before to 3 months after conception | 3 | 0.485 (0.278 – 0.847) |  | 19.98 (p < 0.001) | 89.00% |
|  | 3 months before and continued after conception | 1 | 0.650 (0.401 – 1.053) |  | - | - |
|  | 3 months before conception or first trimester | 1 | 0.540 (0.349 – 0.837) |  | - | - |
|  | 3 months before or 1 month after conception | 1 | 0.481 (0.274 – 0.846) |  | - | - |
|  | Before to 2 months after conception | 1 | 0.585 (0.546 – 0.627) |  | - | - |
|  | Before and continued after conception | 2 | 0.607 (0.531 – 0.695) |  | 1.43 (0.232) | 30.02% |
|  | Before to 3 months after conception | 2 | 1.083 (0.505 – 2.321) |  | 37.52 (p < 0.001) | 97.33% |
| Study locations by world regions | Asia | 9 | 0.606 (0.416 – 0.883) | 11.72 (0.039) | 78.52 (p < 0.001) | 89.81% |
|  | Australia | 3 | 1.034 (0.725 – 1.475) |  | 6.36 (0.042) | 68.57% |
|  | Europe | 11 | 0.654 (0.598 – 0.716) |  | 71.28 (p < 0.001) | 85.97% |
|  | Middle East | 1 | 0.650 (0.401 – 1.053) |  | - | - |
|  | North America | 4 | 0.710 (0.526 – 0.960) |  | 8.07 (0.045) | 62.84% |
|  | South America | 1 | 0.813 (0.706 – 0.937) |  | - | - |
| Food fortification policies | Mandatory | 4 | 0.816 (0.716 – 0.930) | 7.62 (0.055) | 4.57 (0.207) | 34.28% |
|  | Voluntary | 11 | 0.726 (0.635 – 0.830) |  | 114.70 (p < 0.001) | 91.28% |
|  | None | 2 | 0.308 (0.130 – 0.731) |  | 7.91 (0.005) | 87.36% |
|  | Not reported | 12 | 0.691 (0.619 – 0.771) |  | 21.10 (0.032) | 47.87% |

**SUPPLEMENTARY MATERIAL 7: SUB-GROUP ANALYSIS FOR POSTCONCEPTIONAL SUPPLEMENTATION**

| **Study characteristics** | **Sub-groups** | **Number of studies** | **Odds Ratio (95% CI)** | **Between-group difference**  **(p-value)** | **Cochrane’s Q statistic (p-value)** | **I^2^ statistic** |
| --- | --- | --- | --- | --- | --- | --- |
| Overall (postconception) | - | 34 | 0.796 (0.744 – 0.851) | - | 227.82 (p < 0.001) | 85.52% |
| Study design | Case-control | 3 | 0.985 (0.857 – 1.133) | 9.40 (0.009) | 2.40 (0.301) | 16.69% |
|  | Cohort | 8 | 0.711 (0.602 – 0.839) |  | 55.17 (p < 0.001) | 87.31% |
|  | Cross-sectional | 23 | 0.817 (0.764 – 0.874) |  | 71.98 (p < 0.001) | 69.44% |
| Study setting | Hospital-based | 17 | 0.783 (0.681 – 0.901) | 0.08 (0.773) | 42.62 (p < 0.001) | 62.46% |
|  | Population-based | 17 | 0.802 (0.739 – 0.870) |  | 184.33 (p < 0.001) | 91.32% |
| Population | Pregnant women | 24 | 0.776 (0.709 – 0.849) | 0.83 (0.363) | 86.86 (p < 0.001) | 73.52% |
|  | Postpartum women | 10 | 0.825 (0.749 – 0.908) |  | 75.15 (p < 0.001) | 88.02% |
| Method of assessing folic acid intake | Medical records | 3 | 0.709 (0.608 – 0.826) | 2.54 (0.111) | 31.57 (p < 0.001) | 93.67% |
|  | Questionnaire | 31 | 0.813 (0.757 – 0.873) |  | 122.59 (p < 0.001) | 75.53% |
| Trimesters | First trimester | 10 | 0.730 (0.649 – 0.822) | 15.15 (0.001) | 42.58 (p < 0.001) | 78.86% |
|  | Later trimesters | 4 | 0.956 (0.873 – 1.046) |  | 2.22 (0.528) | 0.00% |
|  | Trimester not specified | 20 | 0.788 (0.723 – 0.860) |  | 102.09 (p < 0.001) | 81.39% |
| Study locations by world regions | Africa | 8 | 0.877 (0.710 – 1.083) | 20.23 (0.003) | 27.67 (p < 0.001) | 74.70% |
|  | Asia | 6 | 0.661 (0.507 – 0.862) |  | 20.24 (0.001) | 75.30% |
|  | Australia | 2 | 0.663 (0.558 – 0.788) |  | 1.34 (0.246) | 25.62% |
|  | Europe | 10 | 0.858 (0.782 – 0.941) |  | 22.70 (0.007) | 60.35% |
|  | Middle East | 3 | 0.963 (0.866 – 1.072) |  | 2.31 (0.315) | 13.36% |
|  | North America | 4 | 0.688 (0.442 – 1.070) |  | 21.10 (p < 0.001) | 85.78% |
|  | South America | 1 | 0.787 (0.733 – 0.845) |  | - | - |
| Food fortification policies | Mandatory | 2 | 0.686 (0.663 – 0.709) | 25.54 (p < 0.001) | 0.24 (0.624) | 0.00% |
|  | Voluntary | 6 | 0.827 (0.742 – 0.923) |  | 11.35 (0.045) | 55.97% |
|  | None | 3 | 0.906 (0.767 – 1.070) |  | 1.40 (0.496) | 0.00% |
|  | Not reported | 23 | 0.790 (0.724 – 0.862) |  | 116.34 (p < 0.001) | 81.09% |

**SUPPLEMENTARY MATERIAL 8: FUNNEL PLOTS ASSESSING PUBLICATION BIAS IN STUDIES**


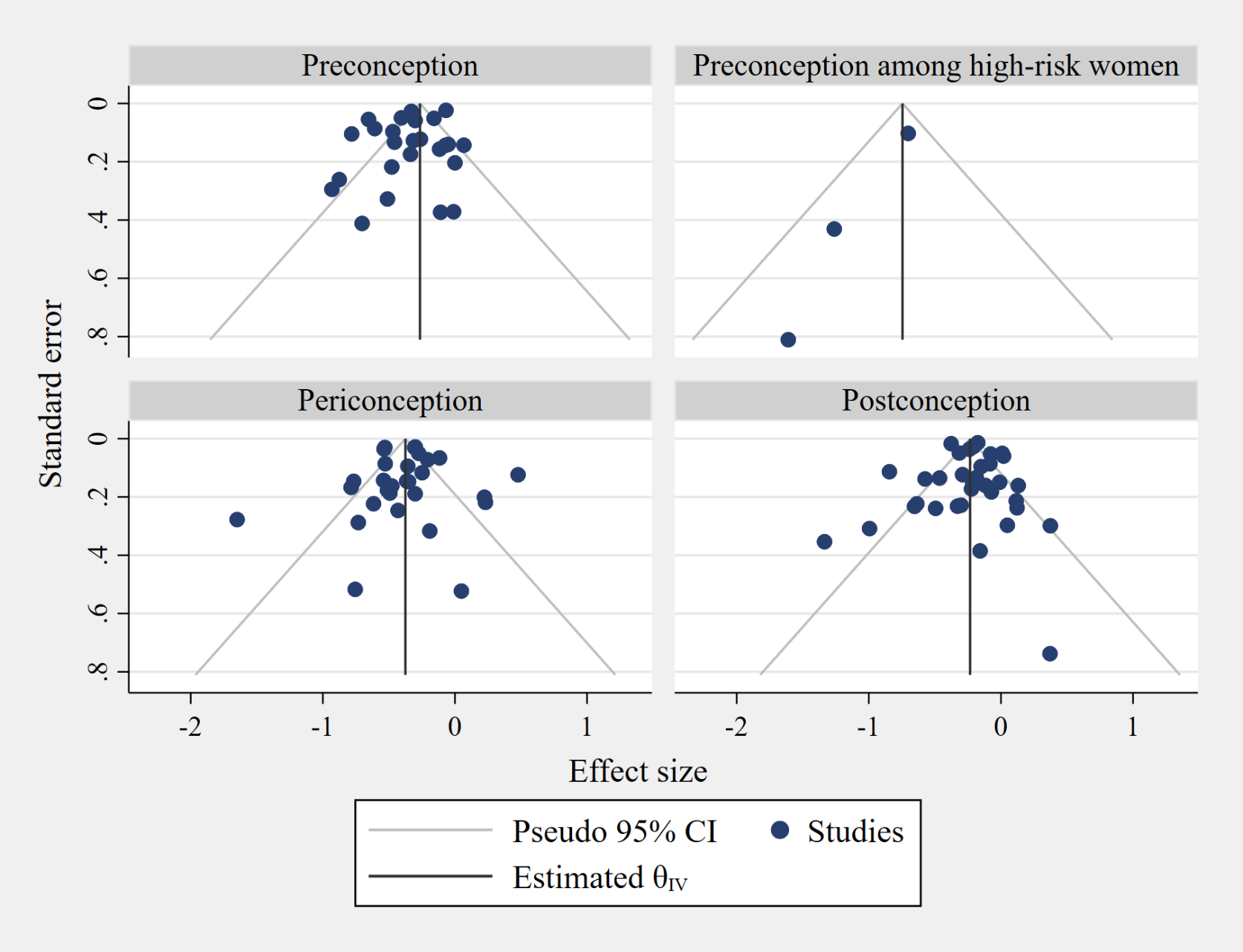

Supplement: online supplemental file 1 [file bmjopen-16-5-s001.docx]
