# Supplementary material for: Does previous pregnancy experience improve folic acid supplementation uptake? A systematic review and meta-analysis
Source: BMJ Open. 2026 May 4;16(5):e106205. doi: 10.1136/bmjopen-2025-106205 (PMC13141133; doi:10.1136/bmjopen-2025-106205)
Supplement: Supplementary file 2 [file bmjopen-2025-106205.reviewer_comments.pdf]

PEER REVIEW HISTORY

BMJ Open publishes all reviews undertaken for accepted manuscripts. Reviewers are asked to complete a checklist review form and are provided with free text boxes to elaborate on their assessment. These free text comments are reproduced below.

ARTICLE DETAILS

Title (Provisional)

Does previous pregnancy experience improve folic acid supplementation uptake? A systematic review and meta-analysis

Authors

Ebong, Utibe; Wilson, Jason; Given, Joanne Emma; Casey, Frank; Loane , Maria; Dolk, Helen

---

VERSION 1 - REVIEW

**Reviewer**	**1**
**Name**	**Lassi, Zohra**
**Affiliation**	**University of Adelaide, School of Public Health**
**Date**	**23-Jul-2025**
**COI**	**None**

Thank you for the opportunity to review this insightful and well-executed manuscript. Overall, this is a valuable contribution to the literature. I commend the authors for conducting a thorough and clearly structured meta-analysis. I recommend major revisions to further improve the clarity and rigour of the manuscript prior to acceptance.

-The abstract notes that data extraction and risk of bias assessment were conducted by a single reviewer. This raises concerns regarding potential bias. Consider acknowledging this limitation more explicitly and, if possible, describe any strategies used to mitigate subjectivity or error.

- The introduction would benefit from improved formatting and sentence structure. Several very short sentences (e.g., lines 25–27) could be linked or revised for better narrative flow.

- In line 21, the statement “serum folate levels of most of the population are insufficient” lacks clarity, please specify the population being referred to (e.g., global, regional, or specific country-level data).

- Consider expanding the introduction to include global data on the prevalence of folic acid or multivitamin (FMC) supplementation. Is usage consistent across different regions, age groups,

or income levels? Is FMC uptake widely known in low- and middle-income countries (LMICs) or mostly confined to high-income countries (HICs)? This would help contextualise your study population and findings.

- It may also be helpful to briefly mention any known risks associated with excessive folic acid intake during pregnancy, to balance the discussion of its benefits.

- The research question appears to be framed as though multiparous women are more likely to use folic acid compared to primiparous women; however, this contradicts both the study's findings and hypothesis. Clarification is needed to ensure alignment between the framing and the study's objectives.

- Line 46: There appears to be a referencing error, which recurs throughout the manuscript. Please review and correct.

- Line 51: Please provide more detail regarding participant recruitment in the "population-based" studies. Were participants recruited via clinics, community centres, advertisements, or other means?

- While the heterogeneity of parity definitions across studies is acknowledged, it remains unclear how this variability was addressed in the synthesis. Further elaboration is warranted.

- The text mentions lower heterogeneity among studies using consistent intake definitions or methodologies, but does not report specific heterogeneity statistics or thresholds (e.g., I^2 values). Including these would improve the robustness of the results interpretation.

- A brief summary of regional differences in FMC usage would enhance the results section, even if this is only descriptive.

- The decision not to pool adjusted odds ratios due to variation in confounders is appropriate; however, it would be useful to list the most commonly adjusted confounders (e.g., maternal age, pregnancy planning).

- The discussion references the Capability–Opportunity–Motivation Behaviour (COM-B) framework and provides preliminary recommendations for improving health service delivery. It would strengthen the discussion to more explicitly map these recommendations back to the COM-B domains. For example, what specific interventions are needed to address motivational barriers? Should there be greater emphasis on health education, workforce training (e.g., for nurses/midwives), or policy changes (e.g., subsidised FMC access)?

- Consider citing existing studies that have implemented COM-B-informed strategies to increase preconception health behaviours.

- The section on postconceptional supplementation could benefit from improved clarity and sentence structure.

- Line 56, page 12: The low prevalence of folic acid supplementation among high-risk women is noted as surprising. It would be helpful if the authors could offer a hypothesis or contextual explanation for this finding.

- A brief discussion of how socioeconomic status or educational level may limit access to FMC in countries without mandatory folate fortification would enhance the equity lens of the analysis.

- Please include a short paragraph outlining recommendations for future research in this area, particularly regarding parity-specific interventions and knowledge gaps in low-resource settings.

- Consider overlaying the relevant numbers or percentages directly onto the map for clearer visual interpretation.

Thank you once again for the opportunity to review this manuscript.

**Reviewer**	**2**
**Name**	**MacGregor, Becky**
**Affiliation**	**University of Birmingham, Applied Health Sciences**
**Date**	**10-Sep-2025**
**COI**	**None**

Thank you for an interesting paper.

The title indicates that the review will look at antenatal care experience, which implies qualitative outcomes, but this has not been addressed in the paper. Would suggest revising this to more accurately reflect the scope of the review. For example 'What effect does parity have on uptake of pre-conceptual and antenatal folic acid supplementation?'

Thank you for including a PRISMA checklist, please could you also include a PRISMA abstract checklist?

Lines 32-38 of the introduction, state that guidelines recommend folic acid from pre-pregnancy to 12 weeks of gestation. Review question looks at use for one month post-conception. Why was a one month time period chosen?

Line 54 of 'Definition of parity...' section. Please re-word this sentence to make it clearer.

Please correct the reference errors in bold throughout the text.

Please consider re-ordering the methods section to have the search strategy come after the review questions, eligibility and exclusion criteria.

Modified Newcastle Ottawa Score was used to assess bias risk which is designed to assess case-control and cohort studies. How were modifications decided on as the categories used seem to differ quite significantly from the standard template found in the link below? Was a separate scoring system used for cohort studies vs case control studies as recommended by

the original tool? 74% of the studies were cross-sectional, why was NOS used to assess these studies?

https://www.ohri.ca/programs/clinical_epidemiology/oxford.asp

Line 17 page 5. Mentions that the effect of parity was not the sole objective in any of the studies. Does this mean the studies had multiple objectives including the effect of parity or that the effect of parity was a secondary objective or that it wasn't an objective in the studies at all? Please clarify.

Line 41-52 page 8. Please consider revising the wording for this section with less definitive language, recognising that the results were unable to be adjusted for confounders.

Line 9 page 9. Please remove the word 'should' and rephrase, for example 'we would expect multiparous women to have better knowledge'.

Lines 29-39 page 9. I am not sure that this conclusion about antenatal care can be drawn based on the data as the review hasn't looked at why there is a disparity only to see if there is a disparity and it hasn't adjusted for confounders. For example, guidelines recommend that FAS is continued until the end of the 1st trimester and this may be a more plausible explanation for the difference seen between 1st and 2nd trimester supplementation than the quality of antenatal care.

Lines 56-58 page 9. Please rephrase this or include a reference to back up the statement. High-risk women may not have more exposure to healthcare professionals than low-risk women and may not to be more motivated than low-risk women.

Lines 12 -23, page 10. Please consider revising this paragraph as it currently implies that the review examined the difference between women who had specialist gynae care vs women who saw their neurologist or GP, but this doesn't appear to be reported in the results section.

Please include in the limitations section that searches were restricted to English Language only.

In both the results section and the discussion effects of maternal age, pregnancy planning and food fortification are mentioned as separate results categories, but the review questions do not address these topics.

**Reviewer**	**3**
**Name**	**Dack, Kyle**
**Affiliation**	**University of Bristol, MRC Integrative Epidemiology Unit**
**Date**	**24-Oct-2025**
**COI**	**None**

Statistical review summary:

The paper is clearly written, and the search methodology adhere to good systematic review practices.

However, the decision to meta-analyse unadjusted estimates introduces substantial risk of confounding bias and undermines the interpretability of the findings. Methodological guidelines such as Cochrane generally recommend pooling estimates even if the covariates differ, as these are still typically more valid than unadjusted results.

The rationale provided for this decision is not well supported, and also leads to an inconsistency with the risk of bias assessment which was based on adjusted results.

The authors should consider using adjusted results, or do both adjusted and unadjusted meta-analyses, to evaluate their impact.

Apart from this, the manuscript is well structured and my remaining comments are minor and relate to clarity.

Abstract:

1. The abstract is clear, contains all required PRISMA elements, and the statistical reporting is easy to follow. I do not have suggestions or queries.

Introduction:

2. As above – no comments or suggestions.

Methods:

3. “The systematic review was conducted in accordance with the PRISMA guidelines” It would be more accurate to state that the manuscript was written in accordance with PRISMA, as PRISMA is a reporting guideline rather than methods guide (that would be Cochrane or similar).

4. I suggest inserting the PROSPERO registration after the first line, and if there is space, a high-level overview of the study (e.g. search dates, outcome of interest, population of interest) to help readers get a general sense of the methods prior to going into the details.

5. Review questions: I suggest this would flow better if placed at the end of the introduction or beginning of the methods, rather than after the search strategy – because the search strategy was determined by the review questions.

6. Risk of bias tool: It is very helpful – and strengthens the transparency of the paper - that the authors provided their study-specific criteria for each NOS item, particularly which confounding factors were required to be adjusted. I see very few reviews which provide this.

7. “Two studies were excluded from the review due to a high risk of bias (Error! Reference source not found.).38,39” The previous line defined the thresholds used to determine studies of moderate quality and high quality, but not high risk of bias. Note there is also a stray error message here.

8. Supplementary Material 4. I suggest changing the phrasing from “low quality” to “high risk of bias”, or vice versa, so that the terminology aligns in all parts of the paper – this will enhance consistency and readability.

9. Stronger justification is needed for the decision to meta-analyse of unadjusted rather than adjusted odds ratios.

-The first rationale provided – that studies adjusted for different covariates – is not supported by methodological guidance. Major sources such as Cochrane do not advice against pooling results adjusted for different covariates. In fact, differences are expected and are the reason statistical heterogeneity and risk of bias are evaluated to judge whether differences impacted the consistency of the results.

-Conversely, there is explicit discouraging of using unadjusted estimates over adjusted estimates, because of the higher risk of confounding bias (Cochrane chapter 24). Moreover, if the intention was to reduce heterogeneity, the results do not seem to support this because the statistical heterogeneity remains high.

-The second justification regarding data availability was not clear to me. I suggest elaborating on both of the justifications to make them stronger, or considering an alternative approach. One approach the authors may wish to consider is adding an extra analysis, including only studies where the adjusted odds ratios were reported, and comparing whether there is meaningful difference between this and the unadjusted pooled estimates.

10. The analysis leads to an inconsistency between the RoB grading and the meta-analysis. The RoB item “comparability” was based on adjusted estimates, but all of the meta-analysis estimates are unadjusted. This means the RoB does not fully correspond to the statistics which were pooled – if all statistics were unadjusted estimates, then all studies should be scored 0 because there was no adjustment for age of pregnancy planning.

11. GRADE: The above issue could also propagate into GRADE, depending on how many studies ratings are changed by the loss of comparability scores.

Results

12. Figure 1. The methods also listed Medline-OVID, was this database searched?

13. “Twenty-eight studies were assessed as having a moderate risk of bias, and 53 as having a low risk of bias” I recommend aligning with the language used in the methods (or vice versa) to avoid any ambiguity.

14. The results are really clear and well written, including the comments which interpret the statistics after reporting them. However, it would help readers (and align with PRISMA) if the number of studies, certainty of evidence (GRADE), and a heterogeneity statistic (e.g. I2) were reported for each meta-analysis, because these have a major impact on how to interpret the findings.

15. “Further analysis (Supplementary Material 6) showed that heterogeneity was lower among studies that used the same methods to assess folic acid intake (medical records, questionnaire,

or both)" similar to the above point I suggest supporting this by reporting the lower I2. The authors could comment on whether the OR remains stable or differs in these groups.

Discussion

16. The discussion is reasonable and well written, but I suggest integrating certainty of evidence into it more, to ensure readers interpret the findings accurately. Currently this is discussed in the limitations section – but GRADE is intended to represent the strength of inference, and is core to the results.

VERSION 1 - AUTHOR RESPONSE

Reviewer 1 Comments

- The abstract notes that data extraction and risk of bias assessment were conducted by a single reviewer. This raises concerns regarding potential bias. Consider acknowledging this limitation more explicitly and, if possible, describe any strategies used to mitigate subjectivity or error.
  Thank you for this comment. Although data extraction was performed by one reviewer, two reviewers independently screened the titles and abstracts for all records returned from the search terms, as well as the full text for eligible studies. In addition, the quality of a random 10% of studies were independently assessed by three other reviewers. We have clarified this in the Abstract and in the Strengths and limitations, as shown below:
  Abstract: "Two reviewers screened all papers meeting the eligibility criteria. One reviewer completed data extraction and assessed study quality using an adapted version of the Newcastle -Ottawa Scale. Three other reviewers independently assessed 10% of the studies as a quality check."

  Strengths and limitations: "While risk of bias assessment was conducted by one reviewer, a random subset (10%) of these studies were independently assessed by three other reviewers to ensure reliability and accuracy."
- The introduction would benefit from improved formatting and sentence structure. Several very short sentences (e.g., lines 25–27) could be linked or revised for better narrative flow.
  The Introduction section has been rewritten to improve clarity and narrative flow (Page 1).
- In line 21, the statement "serum folate levels of most of the population are insufficient" lacks clarity, please specify the population being referred to (e.g., global, regional, or specific country-level data).
  The Introduction section has been rewritten, and this sentence now reads as (page 1): "In a systematic review, Rogers et al. found that the prevalence of folate insufficiency exceeded 40% in most countries surveyed.[17]"
- Consider expanding the introduction to include global data on the prevalence of folic acid or multivitamin (FMC) supplementation. Is usage consistent across different regions, age groups, or income levels? Is FMC uptake widely known in low- and middle-income

countries (LMICs) or mostly confined to high-income countries (HICs)? This would help contextualise your study population and findings.

The Introduction section has now been expanded to include these and other useful contexts (page 1): "Despite supplementation being recommended since the 1990s,[18–22] Toivonen et al. reported variable prevalence of preconceptional folic acid supplementation across countries, with very low prevalence in African studies and higher prevalence among populations with higher educational levels.[23] Within populations, factors such as older maternal age and pregnancy planning are known to increase women's likelihood of taking folic acid supplements preconceptionally.[24–31]"

- It may also be helpful to briefly mention any known risks associated with excessive folic acid intake during pregnancy, to balance the discussion of its benefits.

  In discussing our findings among high-risk women, we signposted to concerns from literature on the side effects due to high dose folic acid (page 10):

  "Indeed, some studies have questioned the benefits of high dose folic acid for high-risk pregnant women compared to the normal (400 mcg) dose.[151,152] The safety of high dose folic acid has also been investigated.[153–155] Nonetheless, there is clear evidence of the importance of early initiation and uptake of folic acid supplements in preventing NTDs, particularly among this high-risk group of women."
- The research question appears to be framed as though multiparous women are more likely to use folic acid compared to primiparous women; however, this contradicts both the study's findings and hypothesis. Clarification is needed to ensure alignment between the framing and the study's objectives.

  The review questions have been rephrased to align with the review's objectives:
  1. Are multiparous women more or less likely to take folic acid in the preconceptional period (at least 1 month before conception) compared to primiparous women?
  2. Are multiparous women more or less likely to take folic acid in the periconceptional period (at least 1 month before conception and continued use for at least 1 month after conception) than primiparous women?
  3. Are multiparous women more or less likely to take folic acid in the postconceptional period (taking supplementation after conception) than primiparous women?
  4. Are multiparous high-risk women (those with epilepsy, pre-gestational diabetes or previous infants born with NTD) more or less likely to take folic acid than primiparous high-risk pregnant women?
- Line 46: There appears to be a referencing error, which recurs throughout the manuscript. Please review and correct.

  Errors in displaying references have been resolved.
- Line 51: Please provide more detail regarding participant recruitment in the "population-based" studies. Were participants recruited via clinics, community centres, advertisements, or other means?

  These included studies in which women were either recruited from communities or their data were taken from national registers or databases. This detail has been included as follows (page 4):

"Regarding study settings, 33 (41%) of these studies were hospital-based, and 48 (59%) were population-based where participants were recruited from communities or national registers/databases."

- While the heterogeneity of parity definitions across studies is acknowledged, it remains unclear how this variability was addressed in the synthesis. Further elaboration is warranted.

  The review used parity as defined in the included studies; no analysis was conducted on this, as it was not possible to determine exactly what primiparous or multiparous meant across all included studies. This has been elaborated as follows:

  Definition of parity and gestational timing of FAS section under Methods (page 3):

  "Parity is usually defined based on clinical thresholds for pregnancy viability, where a pregnancy is considered to be viable after 20 gestational weeks.[49] However, many of the studies reviewed did not specify their parity definition or referred to previous pregnancies rather than parity. This review used parity as defined in the included studies."

  We have also acknowledged this as a limitation:

  "Further, this review used as parity as defined by the included studies; however, it is possible that study definitions do not align with existing clinical definitions of parity.[49,178] This meant that in some studies, multiparous women may have included women whose previous pregnancy(ies) did not attain viability. This is important in the context of experiencing full antenatal care and folic acid knowledge/use in their previous pregnancy(ies)."

- The text mentions lower heterogeneity among studies using consistent intake definitions or methodologies, but does not report specific heterogeneity statistics or thresholds (e.g., I^2 values). Including these would improve the robustness of the results interpretation.

  Heterogeneity statistics (Cochrane's Q statistics and I^2 values) and their interpretation have now been included in the Results for each review outcome. An example is shown below:

  Page 7: "Twenty-nine studies examined periconceptional FAS.[24,26–28,30,31,39,41,70,71,73–78,99–111] Pooling these studies, the odds of supplementation were 21% lower among multiparous women (aOR 0.69, 95% CI 0.64 to 0.76, low certainty of evidence) than primiparous women. The absolute effect of multiparity on periconceptional intake was a difference of 7 per 100 women compared to primiparous women (Table 3).

  Heterogeneity was high among these 29 studies (Cochrane's Q test = 194.61, $p < 0.001$, I2 = 85.61%). Heterogeneity was reduced when studies were grouped by food fortification policies (mandatory fortification: aOR 0.82, 95% CI 0.72 to 0.93, Cochrane's Q test = 4.57, p = 0.21, I2 = 34.28%, n = 4, and fortification policy not reported: aOR 0.69, 95% CI 0.62 to 0.77, Cochrane's Q test = 21.10, p = 0.03, I2 = 47.87%, n = 12). The test for differences between these fortification subgroups indicated borderline differences in the pooled ORs (test of group difference = 7.62, p = 0.05; Supplementary Material 6)"

- A brief summary of regional differences in FMC usage would enhance the results section, even if this is only descriptive.

  Thank you for the valuable feedback. We agree that regional differences in FMC usage are important. However, this requires an in-depth assessment of the methods of folic acid

supplementation ascertainment. Our review focused specifically on the parity effect on supplementation rather than prevalence or regional comparisons. There are systematic reviews that examined prevalence or regional comparisons in depth. For example:

  - Aweke et al. (2025): Folic acid supplementation during preconception period in sub-Saharan African countries: A systematic review and meta-analysis
  - Loperfido et al. (2025): Folic acid supplementation in European women of reproductive age and during pregnancy with excessive weight: a systematic review
  - Toivonen et al. (2018): Folic acid supplementation during the preconception period: A systematic review and meta-analysis

- The decision not to pool adjusted odds ratios due to variation in confounders is appropriate; however, it would be useful to list the most commonly adjusted confounders (e.g., maternal age, pregnancy planning).

  Thank you for this useful comment. The frequently adjusted confounders have been listed in the Results for each review outcome:

  Page 6: Preconceptional uptake – maternal age, education, socioeconomic status, smoking, relationship status and pregnancy planning.

  Page 7: Periconceptional uptake – maternal age, education, socioeconomic status, smoking, relationship status and pregnancy planning.

  Page 7: Postconceptional uptake – maternal age, education, socioeconomic status, smoking, relationship status, and place of residence (urban vs rural).

  Page 8: Preconceptional uptake among high-risk women – maternal age, smoking and pregnancy planning

- The discussion references the Capability–Opportunity–Motivation Behaviour (COM-B) framework and provides preliminary recommendations for improving health service delivery. It would strengthen the discussion to more explicitly map these recommendations back to the COM-B domains. For example, what specific interventions are needed to address motivational barriers? Should there be greater emphasis on health education, workforce training (e.g., for nurses/midwives), or policy changes (e.g., subsidised FMC access)?

  Thank you for the feedback. The suggested interventions have now been mapped to the COM-B domains in the 'Implications for clinicians and policymakers' section (page 11): "Preconception care before the first pregnancy, antenatal care during the first pregnancy, and interconception care after the first pregnancy could look ahead to later pregnancies, emphasising the importance of initiating FAS preconceptionally.[162–164] This could strengthen women's capability to supplement periconceptionally. Consideration could be given to the role of the partner and other social support,[159,161] including community-based support groups, to enable the required social opportunity for periconceptional supplementation. In terms of physical opportunity, policy changes such as provision of free folic acid supplements and using a more diverse distribution network,[140,165,166] may improve FAS uptake. Negative beliefs and perceptions can limit women's motivation to change behaviour,[139,140] hence it is important that health information provided by clinicians and others address known misconceptions about FAS to improve women's capability and motivation. The implications for workforce training and the need to include

time for folic acid-related interventions in preconception, antenatal and interconception care need to be considered”

- Consider citing existing studies that have implemented COM-B-informed strategies to increase preconception health behaviours.
  We have now included this on page 11:
  “While the COM-B framework has been used to guide behaviour change interventions for pregnant women in other areas such as smoking,[164] physical activity[141,165] and diet,[140,166] only one study has applied it to folic acid.[142]”
- The section on postconceptional supplementation could benefit from improved clarity and sentence structure.
  This section on postconceptional supplementation has been rewritten (page 10):
  “Evidence from this review shows that multiparous women, overall, had significantly lower odds of taking folic acid postconceptionally, compared to primiparous women. However, by the second and third trimesters, there was no significant difference in uptake between primiparous and multiparous women. By the second trimester, it is expected that almost all pregnant women will have begun antenatal appointments, during which the need for supplementation would be emphasised. This may explain why the supplementation rates between primiparous and multiparous women were similar at this stage of the pregnancy. However, these women would have missed the critical window for supplementation needed to protect against NTDs.”
- Line 56, page 12: The low prevalence of folic acid supplementation among high-risk women is noted as surprising. It would be helpful if the authors could offer a hypothesis or contextual explanation for this finding.
  The WHO has highlighted guidelines for specialised care for high-risk pregnancies, including adequate FA supplementation to prevent NTDs. Studies have found that in practice, not all high-risk pregnancies receive the recommended care due to a myriad of issues. This sentence now reads as:
  Page 10: “The low prevalence in this high-risk group is surprising, as these women would be expected to have more exposure to specialised care as per WHO guidelines.[156] Studies have highlighted gaps in the recommended specialised care for high-risk pregnancies.[157,158]”
- A brief discussion of how socioeconomic status or educational level may limit access to FMC in countries without mandatory folate fortification would enhance the equity lens of the analysis.
  Following comments about the relevance of food fortification to the review questions, we have now removed this section from the results and discussion. As a result, the suggested change is not applicable.
- Please include a short paragraph outlining recommendations for future research in this area, particularly regarding parity-specific interventions and knowledge gaps in low-resource settings.
  A paragraph has been added to the Conclusion highlighting recommendations for future research (page 13): “Future research should explore interventions to promote preconceptional supplementation using the COM-B model of behaviour change, which recognises capability, motivation and opportunity as essential interacting components.”

- Consider overlaying the relevant numbers or percentages directly onto the map for clearer visual interpretation.
  Together with addressing the comments of Reviewer 2 regarding naming the countries, we have tried different methods of labelling, but find that they do not work well for countries of small surface areas. Instead, we have put a clear note on the map that the reader can find the country-specific data in Supplementary Material 2.

Reviewer 2 Comments

- The title indicates that the review will look at antenatal care experience, which implies qualitative outcomes, but this has not been addressed in the paper. Would suggest revising this to more accurately reflect the scope of the review. For example 'What effect does parity have on uptake of pre-conceptual and antenatal folic acid supplementation?'
  Thank you for this feedback. Antenatal care has been removed from the title which now reads: "Does previous pregnancy experience improve folic acid supplementation uptake? A systematic review and meta-analysis"
- Thank you for including a PRISMA checklist, please could you also include a PRISMA abstract checklist?
  Thank you for flagging this; a PRISMA abstract checklist has now been uploaded.
- Lines 32-38 of the introduction, state that guidelines recommend folic acid from pre-pregnancy to 12 weeks of gestation. Review question looks at use for one month post-conception. Why was a one month time period chosen?
  The guideline quoted referred to the UK, but guidelines regarding timing vary across countries. In addition, the timing for "periconception" also varies between studies as shown in Table 1. We used the 1 month minimum time period because it was the most inclusive. This has now been made clear in the manuscript (page 1):
  "Women in the UK who may become pregnant, are planning to become pregnant, or are within the first 12 weeks of pregnancy are advised to take 400 mcg of folic acid each day.[15]"
- Line 54 of 'Definition of parity...' section. Please re-word this sentence to make it clearer.
  This has been clarified as follows:
  Definition of parity and gestational timing of FAS section under Methods (page 3):
  "Parity is usually defined based on clinical thresholds for pregnancy viability, where a pregnancy is considered to be viable after 20 gestational weeks.[49] However, many of the studies reviewed did not specify their parity definition or referred to previous pregnancies rather than parity. This review used parity as defined in the included studies."
- Please correct the reference errors in bold throughout the text.
  Errors in displaying references have been resolved.
- Please consider re-ordering the methods section to have the search strategy come after the review questions, eligibility and exclusion criteria.
  Thank you for the suggestion. The review questions have been moved to the end of the Introduction section. The search strategy has been moved to come after the eligibility and exclusion criteria.

- Modified Newcastle Ottawa Score was used to assess bias risk which is designed to assess case-control and cohort studies. How were modifications decided on as the categories used seem to differ quite significantly from the standard template found in the link below? Was a separate scoring system used for cohort studies vs case control studies as recommended by the original tool? 74% of the studies were cross-sectional, why was NOS used to assess these studies? https://protect.checkpoint.com/v2/r02/___https://www.ohri.ca/programs/clinical_epidemiology/oxford.asp___.YzJlOnVsc3RlcnVuaXZlcnNpdHk6YzpvOjdmZTA3OTZjYmI1ZjM2OGIzNTk2N2ZkNTQ0OTEyODAyOjc6YWEzNjozMzdmZjE5ZTQ3OTI4ZjkyMmZiNGUxN2YzODZjNGYyM2E1MDhhMDljNWJhYTg4N2E0ZjJjNzRkYTU4MjM0YTYzOnQ6RjpO
  The choice of using the NOS tool for assessing these studies was based on its suitability when compared to other tools, such as the ROBINS-I, etc. We adapted the tool, retaining domains that were applicable to our review, such as 'Assessment of outcome' (which we renamed 'Measurement of outcome'), and 'Comparability', where we scored for the adjusting of pregnancy planning and maternal age; two key confounders in the association between FAS and parity. We removed domains not applicable to the scope of our review, such as 'Is the case definition adequate?' 'Selection of Controls', and ' Definition of Controls', since our review was not centred on cases of a disease outcome.
  Previous systematic reviews have adapted the NOS tool to cohort and cross-sectional studies, meaning that adapting the tool is an established approach:
  (1) Orton S, Coleman T, Coleman-Haynes T, Ussher, M. Predictors of Postpartum Return to Smoking: A Systematic Review, Nicotine & Tobacco Research, Volume 20, Issue 6, June 2018, Pages 665–673, https://doi.org/10.1093/ntr/ntx163.
  (2) Herzog, R., Álvarez-Pasquin, M.J., Díaz, C. et al. Are healthcare workers' intentions to vaccinate related to their knowledge, beliefs and attitudes? a systematic review. BMC Public Health 13, 154 (2013). https://doi.org/10.1186/1471-2458-13-154
- Line 17 page 5. Mentions that the effect of parity was not the sole objective in any of the studies. Does this mean the studies had multiple objectives including the effect of parity or that the effect of parity was a secondary objective or that it wasn't an objective in the studies at all? Please clarify.
  For clarity, this sentence has been revised as follows (page 5): "Parity was examined as one of the many sociodemographic determinants of folic acid uptake in some of the included studies. In others, it was merely reported as one of the analysis outputs, but its effect was not discussed."
- Line 41-52 page 8. Please consider revising the wording for this section with less definitive language, recognising that the results were unable to be adjusted for confounders.
  Thank you for this comment. Following Reviewer 3's comment on the need to include adjusted ORs, our meta-analysis now includes adjusted ORs. Thus, our conclusions are warranted.
- Line 9 page 9. Please remove the word 'should' and rephrase, for example 'we would expect multiparous women to have better knowledge'.
  The Discussion section has been rewritten and this sentence deleted (page 9).

- Lines 29-39 page 9. I am not sure that this conclusion about antenatal care can be drawn based on the data as the review hasn't looked at why there is a disparity only to see if there is a disparity and it hasn't adjusted for confounders. For example, guidelines recommend that FAS is continued until the end of the 1st trimester and this may be a more plausible explanation for the difference seen between 1st and 2nd trimester supplementation than the quality of antenatal care.
  Although guidelines recommend FA use until the end of the first trimester, studies show that women continue to take folic acid throughout pregnancy [Branum, Bailey and Singer, 2013: https://www.sciencedirect.com/science/article/pii/S0022316622011506?via%3Dihub#s0025, and Holmes et al. 2005: https://academic.oup.com/clinchem/article/51/3/629/5629609?login=true#200033304]
  We were not implying that the quality of care was responsible for the difference in uptake between the first and second trimesters; rather, by the second trimester, we would expect that almost all pregnant women would have been exposed to antenatal care, where emphasis on FA supplementation would be made, thus prompting more women initiate uptake.
  This section on postconceptional uptake now reads as (page 10):
  "Evidence from this review shows that multiparous women, overall, had significantly lower odds of taking folic acid postconceptionally, compared to primiparous women. However, by the second and third trimesters, there was no significant difference in uptake between primiparous and multiparous women. By the second trimester, it is expected that almost all pregnant women will have begun antenatal appointments, during which the need for supplementation would be emphasised. This may explain why the supplementation rates between primiparous and multiparous women were similar at this stage of the pregnancy. However, these women would have missed the critical window for supplementation needed to protect against NTDs."
- Lines 56-58 page 9. Please rephrase this or include a reference to back up the statement. High-risk women may not have more exposure to healthcare professionals than low-risk women and may not to be more motivated than low-risk women.
  A fine point, thank you. We have rephrased this to read as follows (page 10):
  "The low prevalence in this high-risk group is surprising, as these women would be expected to have more exposure to specialised care as per WHO guidelines.[156]"
- Lines 12 -23, page 10. Please consider revising this paragraph as it currently implies that the review examined the difference between women who had specialist gynae care vs women who saw their neurologist or GP, but this doesn't appear to be reported in the results section.
  These sentences referred to the cited studies (Hao et al. 2015 and Herzog et al. 2017), not our review findings.
- Please include in the limitations section that searches were restricted to English Language only.
  Thank you for flagging this. We have added this to the limitations.

- In both the results section and the discussion effects of maternal age, pregnancy planning and food fortification are mentioned as separate results categories, but the review questions do not address these topics.
  Thank you for flagging these. These sections have been removed from the Results and Discussion as they were not part of the review questions.

Reviewer 3 Comments

- The paper is clearly written, and the search methodology adhere to good systematic review practices. However, the decision to meta-analyse unadjusted estimates introduces substantial risk of confounding bias and undermines the interpretability of the findings. Methodological guidelines such as Cochrane generally recommend pooling estimates even if the covariates differ, as these are still typically more valid than unadjusted results. The rationale provided for this decision is not well supported, and also leads to an inconsistency with the risk of bias assessment which was based on adjusted results. The authors should consider using adjusted results, or do both adjusted and unadjusted meta-analyses, to evaluate their impact. Apart from this, the manuscript is well structured and my remaining comments are minor and relate to clarity.
- Abstract:
  - The abstract is clear, contains all required PRISMA elements, and the statistical reporting is easy to follow. I do not have suggestions or queries.
    No actions required.
- Introduction:
  - As above – no comments or suggestions.
    No actions required.
- Methods:
  - "The systematic review was conducted in accordance with the PRISMA guidelines" It would be more accurate to state that the manuscript was written in accordance with PRISMA, as PRISMA is a reporting guideline rather than methods guide (that would be Cochrane or similar).
    The sentence has been revised to read as (page 4): "The review was reported in accordance with PRISMA guidelines".
  - I suggest inserting the PROSPERO registration after the first line, and if there is space, a high-level overview of the study (e.g. search dates, outcome of interest, population of interest) to help readers get a general sense of the methods prior to going into the details.
    The PROSPERO registration number has been included in the introductory sentences to the Methods section:
    "The protocol for this review was registered on the International Prospective Register of Systematic Review (PROSPERO), with registration number CRD42024553241."
- Review questions: I suggest this would flow better if placed at the end of the introduction or beginning of the methods, rather than after the search strategy – because the search strategy was determined by the review questions.

Thank you, the review questions have been moved to the end of the Introduction section.

- Risk of bias tool: It is very helpful – and strengthens the transparency of the paper - that the authors provided their study-specific criteria for each NOS item, particularly which confounding factors were required to be adjusted. I see very few reviews which provide this.

  No actions required.

- “Two studies were excluded from the review due to a high risk of bias (Error! Reference source not found.).38,39” The previous line defined the thresholds used to determine studies of moderate quality and high quality, but not high risk of bias. Note there is also a stray error message here.

  The risk of bias categories have now been uniformly named as low/moderate/high quality to ensure consistency throughout the manuscript and supplementary materials. Also, referencing errors have been fixed.

- Supplementary Material 4. I suggest changing the phrasing from “low quality” to “high risk of bias”, or vice versa, so that the terminology aligns in all parts of the paper – this will enhance consistency and readability.

  The risk of bias categories have now been uniformly named as low/moderate/high quality to ensure consistency throughout the manuscript and supplementary materials.

- Stronger justification is needed for the decision to meta-analyse of unadjusted rather than adjusted odds ratios. The first rationale provided – that studies adjusted for different covariates – is not supported by methodological guidance. Major sources such as Cochrane do not advice against pooling results adjusted for different covariates. In fact, differences are expected and are the reason statistical heterogeneity and risk of bias are evaluated to judge whether differences impacted the consistency of the results. Conversely, there is explicit discouraging of using unadjusted estimates over adjusted estimates, because of the higher risk of confounding bias (Cochrane chapter 24). Moreover, if the intention was to reduce heterogeneity, the results do not seem to support this because the statistical heterogeneity remains high. The second justification regarding data availability was not clear to me. I suggest elaborating on both of the justifications to make them stronger, or considering an alternative approach. One approach the authors may wish to consider is adding an extra analysis, including only studies where the adjusted odds ratios were reported, and comparing whether there is meaningful difference between this and the unadjusted pooled estimates. The analysis leads to an inconsistency between the RoB grading and the meta-analysis. The RoB item “comparability” was based on adjusted estimates, but all of the meta-analysis estimates are unadjusted. This means the RoB does not fully correspond to the statistics which were pooled – if all statistics were unadjusted estimates, then all studies should be scored 0 because there was no adjustment for age of pregnancy planning.

  Thank you for the detailed feedback. Where studies report both unadjusted and adjusted ORs, we have now prioritised the adjusted OR in line with the Cochrane handbook and

your comments. The meta-analysis is now based on adjusted ORs, where adjusted ORs were available, and unadjusted ORs, where only unadjusted ORs were reported.
We have now included sensitivity analysis using only adjusted ORs for each outcome, in order to compare these to the main analysis.
In order to allow the meta-analysis of adjusted estimates, we also expanded the methodology to include the inverse-variance weighting method, which allowed us to combine subgroup adjusted odds ratios from 12 studies that reported multiple subgroups of multiparous women rather than a single multiparous group (this approach is explained in the Methods section, page 4). This resulted in more studies being included in the meta-analysis (81) than in the original manuscript (69).

- GRADE: The above issue could also propagate into GRADE, depending on how many studies ratings are changed by the loss of comparability scores.

  Thank you for this feedback. We have now included adjusted odds ratios as mentioned above. Here is an example of a result statement:
  Page 6: "Twenty-five studies examined preconceptional FAS.[25,29,42,44–46,67,81–98] Pooling these preconceptional studies, the odds of supplementation were 29% lower among multiparous women (aOR 0.71; 95% CI 0.64 to 0.79; low certainty of evidence) compared to primiparous women. The absolute effect of multiparity on preconceptional intake was a difference of 8 per 100 women compared to primiparous women (Table 3). Significant heterogeneity was observed across these 25 studies (Cochrane's Q test = 211.75, $p < 0.001$, $I^2 = 88.67\%$). Heterogeneity was lower when studies were grouped by study locations (studies conducted in Australia: OR 0.83, 95% CI 0.56 to 1.21, Cochrane's Q test = 1.50, p = 0.22, $I^2 = 33.24\%$, n = 2, and studies conducted in the Middle East: OR 0.74, 95% CI 0.55 to 1.02, Cochrane's Q test = 0.31, p = 0.58, $I^2 = 0.00\%$, n = 2). The difference in pooled ORs between these study location subgroups was not significant (test of group difference = 9.80, p = 0.08; Supplementary Material 5).
  Twenty-three of the 25 studies adjusted for confounders, most frequently maternal age, education, socioeconomic status, smoking, relationship status and pregnancy planning. A sensitivity analysis restricted to these 23 studies found that the odds were 31% lower among multiparous than primiparous women (aOR 0.69, 95% CI 0.62 to 0.77, n = 23 studies, $I^2 = 89.44\%$)."
- Results
  - Figure 1. The methods also listed Medline-OVID, was this database searched?
    Yes, four databases were searched, including Medline-OVID (the other three were CINAHL Ultimate, SCOPUS, and ProQuest Medical)
  - "Twenty-eight studies were assessed as having a moderate risk of bias, and 53 as having a low risk of bias" I recommend aligning with the language used in the methods (or vice versa) to avoid any ambiguity.

    The risk of bias categories have now been uniformly named as low/moderate/high quality to ensure consistency throughout the manuscript and supplementary materials. Further, since our Results are now based on 81 studies with adjusted ORs as well as

those with only unadjusted ORs (as explained above); thus, the risk of bias assessment was also revised to reflect 81 studies (page 6):
"Of the 81 studies, 30 studies were assessed as having moderate quality, and 51 as having high quality (Supplementary Material 4)."

The results are really clear and well written, including the comments which interpret the statistics after reporting them. However, it would help readers (and align with PRISMA) if the number of studies, certainty of evidence (GRADE), and a heterogeneity statistic (e.g. I2) were reported for each meta-analysis, because these have a major impact on how to interpret the findings.

Thank you for pointing this out. For each outcome, we have now included these details. An example is shown below:
Page 6: Twenty-five studies examined preconceptional FAS.[25,29,42,44–46,67,81–98]
Pooling these preconceptional studies, the odds of supplementation were 29% lower among multiparous women (aOR 0.71; 95% CI 0.64 to 0.79; low certainty of evidence) compared to primiparous women. The absolute effect of multiparity on preconceptional intake was a difference of 8 per 100 women compared to primiparous women (Table 3).
Significant heterogeneity was observed across these 25 studies (Cochrane's Q test = 211.75, $p < 0.001$, $I^2 = 88.67\%$)."

- "Further analysis (Supplementary Material 6) showed that heterogeneity was lower among studies that used the same methods to assess folic acid intake (medical records, questionnaire, or both)" similar to the above point I suggest supporting this by reporting the lower I2. The authors could comment on whether the OR remains stable or differs in these groups.

  In addition to stating the I^2 value as previously shown, we have further clarified heterogeneity for each review outcome. An example is shown below (page 6):
  "Significant heterogeneity was observed across these 25 studies (Cochrane's Q test = 211.75, p < 0.001, I2 = 88.67%). Heterogeneity was lower when studies were grouped by study locations (studies conducted in Australia: OR 0.83, 95% CI 0.56 to 1.21, Cochrane's Q test = 1.50, p = 0.22, I2 = 33.24%, n = 2, and studies conducted in the Middle East: OR 0.74, 95% CI 0.55 to 1.02, Cochrane's Q test = 0.31, p = 0.58, I2 = 0.00%, n = 2). The difference in pooled ORs between these study location subgroups was not significant (test of group difference = 9.80, p = 0.08; Supplementary Material 5)."

- Discussion
  - The discussion is reasonable and well written, but I suggest integrating certainty of evidence into it more, to ensure readers interpret the findings accurately. Currently this is discussed in the limitations section – but GRADE is intended to represent the strength of inference, and is core to the results.

Thank you for raising this point. We have incorporated GRADE into our Principal Findings (Discussion section) as shown below (page 9):
"In this review, publication bias was not detected across the assessed outcomes. However, the certainty of evidence was limited by substantial heterogeneity for three of the four outcomes. Only one outcome (preconceptional uptake among high risk women) achieved a moderate level of certainty. While these findings indicate a clear effect of multiparity on folic acid uptake, this should be interpreted with caution due to methodological and clinical differences between the included studies."

We believe that the revisions outlined above address all concerns, and we look forward to BMJ Open's further consideration of our revised submission.

VERSION 2 - REVIEW

**Reviewer**	**2**
**Name**	**MacGregor, Becky**
**Affiliation**	**University of Birmingham, Applied Health Sciences**
**Date**	**04-Feb-2026**
**COI**	

Thank you for addressing the reviewers comments so thoroughly. A couple of very minor points.

Please move the title 'Methods' to above the review questions on page 77.

Page 87 lines 13/14, please rephrase 'despite exposure to available preconception, antenatal and interconception care'. Preconception and interconception care are not common in the UK or worldwide, I would suggest changing to 'despite exposure to previous antenatal care.' as this is the only thing you can be fairly certain multiparous women have been exposed to.

Page 90 lines 3/4/5 - consider including that currently very few women receive preconception or interconception care which is the opportunity barrier which comes before emphasising the importance of FAS during appointments.

Page 90 27/28/29/30 - Please remove the word 'should' and re-phrase; whilst it is recommended that high-risk women have preconception care, it is often not happening, so unless we can address why this isn't happening (likely to be multi-faceted and the rate of unplanned pregnancy will be one of these factors) FAS is unlikely to improve.

Page 90 lines 31/32/33. Please consider removing the sentence about women with high BMI, the reference you cited is from 2023. This has been superseded by the 2025 NICE guidance which states 'Reassure anyone with a body mass index (BMI) of 25 kg/m2 or more..... that they do not need to take more than 400 micrograms of folic acid a day unless they have an increased risk of having a baby with a neural tube defect or other congenital malformation.' https://www.nice.org.uk/guidance/ng247/chapter/Recommendations

---

**Reviewer**	**3**
**Name**	**Dack, Kyle**
**Affiliation**	**University of Bristol, MRC Integrative Epidemiology Unit**
**Date**	**15-Feb-2026**
**COI**	

I appreciate the authors thorough response to the original peer review. My sole concern related to the combination of unadjusted and adjusted odds ratios. The authors have now prioritized adjusted odds ratios where possible, and have shown in sensitivity analyses that the difference from including unadjusted estimates was negligible. The interpretation therefore seems supported by the data, with limitations/caveats fully acknowledged, so I do not have further suggestions.

VERSION 2 - AUTHOR RESPONSE

Reviewer 2 Comments

Thank you for addressing the reviewers comments so thoroughly. A couple of very minor points.

Please move the title 'Methods' to above the review questions on page 77.
The title ‘Methods’ has now been moved as suggested. The ‘review questions’ is now the first sub-section under Methods.

- Page 87 lines 13/14, please rephrase 'despite exposure to available preconception, antenatal and interconception care'. Preconception and interconception care are not common in the UK or worldwide, I would suggest changing to 'despite exposure to previous antenatal care.' as this is the only thing you can be fairly certain multiparous women have been exposed to.
  This sentence has been rephrased as suggested, and now reads as: "We found that multiparous women were consistently less likely than primiparous women to take folic acid from preconception to postconception, despite exposure to previous antenatal care."
- Page 90 lines 3/4/5 - consider including that currently very few women receive preconception or interconception care which is the opportunity barrier which comes before emphasising the importance of FAS during appointments.
  Thank you for this point. We have now prefaced this sentence within the context of low utilisation of preconception and interconception care as the first barrier faced by women:
  "Meta-analyses show low utilisation of preconception[162,163] and interconception care[164], thereby creating the first barrier to improvements in these COM-B components. Preconception care before the first pregnancy, antenatal care during the first pregnancy, and interconception care after the first pregnancy could look ahead to subsequent pregnancies, emphasising the importance of initiating FAS preconceptionally.[165–167]"
- Page 90 27/28/29/30 - Please remove the word 'should' and re-phrase; whilst it is recommended that high-risk women have preconception care, it is often not happening, so unless we can address why this isn't happening (likely to be multi-faceted and the rate of unplanned pregnancy will be one of these factors) FAS is unlikely to improve.
  It is true that high-risk women do not have the expected level of care, including preconception care. This sentence has now been rephrased as follows:
  "For women with conditions such as diabetes and epilepsy, preconception care is recommended to ensure that the condition and treatment are regulated before pregnancy,[170–173] folic acid supplementation could be improved among this high-risk group if preconception care is optimally designed, available and utilised.[162]"
- Page 90 lines 31/32/33. Please consider removing the sentence about women with high BMI, the reference you cited is from 2023. This has been superseded by the 2025 NICE guidance which states 'Reassure anyone with a body mass index (BMI) of 25 kg/m2 or more..... that they do not need to take more than 400 micrograms of folic acid a day unless they have an increased risk of having a baby with a neural tube defect or other congenital malformation.'
  https://www.nice.org.uk/guidance/ng247/chapter/Recommendations___.YzJlOnVsc3RlcnVuaXZlcnNpdHk6YzpvOjc2MjMxNWEwYzJjNzQ2MTgyNGY2M2QzY2U1MzEzZWI4Ojc6NjUyYjphNWYwMjI3YWM4N2Q5NGJiNzdmODk4YmMzMjRiYmUzMzY5NWEwYmZjNzYzZjRiMTM2NzU3Zjc1Y2M3OWYyYzQwOnQ6RjpO
  Thank you for flagging this. The sentence has been removed.

Reviewer 3 Comments

- I appreciate the authors thorough response to the original peer review. My sole concern related to the combination of unadjusted and adjusted odds ratios. The authors have now prioritized adjusted odds ratios where possible, and have shown in sensitivity analyses that the difference from including unadjusted estimates was negligible. The interpretation therefore seems supported by the data, with limitations/caveats fully acknowledged, so I do not have further suggestions.
  No action required, thank you again for your thorough review

We believe that the revisions outlined above address all concerns, and we look forward to BMJ Open's further consideration of our revised submission.

Regards,
Utibe Ebong (on behalf of all authors).
